# Antibacterial, antibiofilm, and antioxidant activities of two novel metal–organic frameworks (MOFs) based on 4,6-diamino-2-pyrimidinethiol with Zn and Co metal ions as coordination polymers†

**DOI:** 10.1039/d4ra00545g

**Published:** 2024-03-18

**Authors:** Rebaz F. Hamarawf

**Affiliations:** a Department of Chemistry, College of Science, University of Sulaimani Kirkuk Road Sulaymaniyah City 46001 Kurdistan Region Iraq rebaz.hamarawf@univsul.edu.iq; b Department of Medical Laboratory Science, Komar University of Science and Technology (KUST) Qliasan St Sulaymaniyah City 46002 Kurdistan Region Iraq

## Abstract

In the present era, the increase in free radical species (FRs) and multidrug-resistant (MDR) bacteria represents a major worldwide concern for public health. Biofilm development and the overuse and misuse of antibiotics could lead to the adaptation of bacteria to antimicrobial agents. Consequently, finding novel multifunctional species with antibacterial, antioxidant, and antibiofilm properties has become crucial in the fight against challenging bacterial infections and chronic inflammatory conditions. Metal–organic frameworks (MOFs) with zinc and cobalt metal centers are widely utilized in biological and environmental remediation owing to their versatility. In this study, multifunctional Zn-MOFs and Co-MOFs were successfully synthesized with zinc and cobalt as metal centers and 4,6-diamino-2-pyrimidinethiol as an organic linker using a hydrothermal technique. Numerous characterization techniques were used to fully examine the MOF structure, functionality, chemical makeup, crystalline structure, surface appearance, thermal behavior, and magnetic characteristics; the techniques included XPS, PXRD, FTIR, FESEM, EDX, UV-visible, BET, BJH, TGA/DTG, DSC, and magnetic susceptibility measurement. The antioxidant, antibacterial, and antibiofilm activities of the MOFs were examined, and they demonstrated potent activity in each of these aspects. The proposed mechanisms of antibacterial activity suggest that bacterial cell death results from multiple toxic effects, including electrostatic interaction and lipid peroxidation, when MOFs are attached to bacteria, leading to the formation of reactive oxygen species (ROSs). Zn-MOFs exhibit high antibacterial and antibiofilm efficacy owing to their large surface-to-volume ratio and porous nature, while Co-MOFs exhibit high antioxidant capacity owing to their redox properties.

## Abstract 2

Two novel metal–organic frameworks (MOFs), synthesized from 4,6-diamino-2-pyrimidinethiol with Zn and Co metal ions, display antibacterial, antibiofilm, and antioxidant properties, serving multifunctionality in biological and environmental domains.

## Graphical abstract

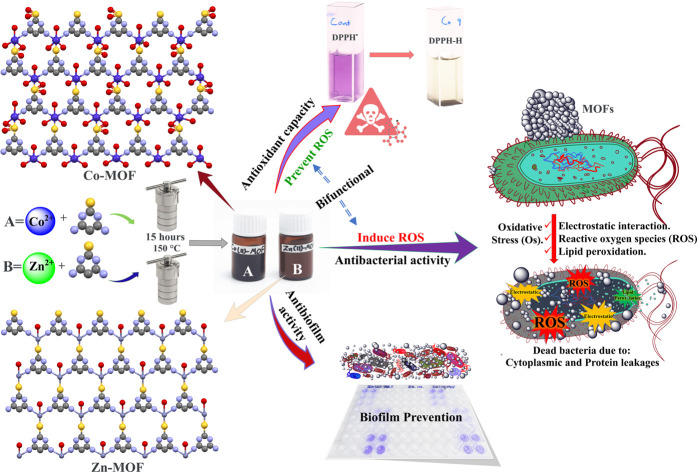

## 1. Introduction

Metal–organic frameworks (MOFs) are porous materials formed by coordinating metal ions or clusters of ions with organic linkers.^1^ Owing to their diversity, tunability, and versatile properties, MOFs have garnered significant attention from researchers in various sectors, including environmental and biomedical fields.^2,3^ They provide an alternative material for fighting against both FRs and bacterial species, boosting their potential as antibacterial agents.^4,5^ In recent times, there has been an increase in the spread of bacterial infectious diseases, including worldwide medical issues resulting from superbugs.^6^ MOFs can be utilized as antibacterial agents or to control the release of a drug to tackle this issue.^7^ Nowadays, nanoparticles (NPs), including those composed of Ag and transition metals such as Fe, Co, Ni, Cu, and Zn, have gained popularity as alternatives to conventional antimicrobial agents.^8–11^ The uncontrolled release of metal ions from metallic NPs can be problematic, potentially causing harm to both bacteria and normal tissues.^12^ To address this issue, researchers are working on preventing such release by encapsulating metal ions within the structure of metal oxide NPs and MOFs.^12,13^ MOFs play a crucial role in catalytic activity because of their notable characteristics, such as high porosity, large surface area, tunable chemical composition, diverse functions, and inherent biodegradability.^14^ These materials have been applied in biomedical and environmental sectors, including chemical sensing,^15^ bio-imaging,^16^ drug delivery,^17^ their uses against FRs and bacterial species,^18^ designing nanozymes,^19^ and environmental remediation such as water purification.^20^ Based on their applications and uses, MOFs can be categorized based on different criteria, such as the characteristics of their metal ions, the type of organic linkers, porosity, functionality, topology, and dimensionality of their network. For instance, some MOFs can have different metal nodes, such as transition metals of zinc,^18^ copper,^4^ and cobalt,^21^ or some have lanthanides,^22^ and actinides,^23^ which can affect their stability, reactivity, and magnetic properties. Some can also have carboxylate,^24^ pyridyl,^25^ or imidazolate as organic linkers,^26^ which can influence their flexibility, polarity, selectivity, catalytic, and sensing efficacies. A few examples of MOFs are porous coordination polymers (PCPs)^27^ and zeolitic imidazolate frameworks (ZIFs).^14^ These are two types of MOFs that have flexible structures that can undergo structural changes in response to various stimuli, including temperature, pressure, and adsorption properties. ZIFs are formed from metal ions of Zn^2+^ or Co^2+^ and imidazolate as ligands,^14^ while PCPs are polymeric structures synthesized by the coordination of metal ions with flexible ligands.^27^ They can have different network topologies and dimensionalities, including cubic, hexagonal, tetragonal, one-dimensional, two-dimensional, or three-dimensional network topologies.^28^ Additionally, MOFs may exhibit features related to micropores (<2 nm), mesopores (2–50 nm), or macropores (>50 nm) depending on the various pore diameters.^29^ They exhibit antibacterial properties that can be used directly or as carriers of antibacterial drugs.^14^ In fact, the antibacterial effect of MOFs may be attributed to several processes, such as their interaction with bacterial cell membranes, which allow them to pass through and cause permeabilization and destabilization of the membrane.^14^ This disruption can result in the leakage of cellular contents, such as protein, the loss of ion gradients, such as potassium, and, ultimately, cell death.^4^ Oxidative stress (Os) is induced *via* superoxide (O_2_˙^−^) and hydroxy radicals (˙OH) as a class of reactive oxygen species (ROS).^30^ These two FRs play a significant role and can directly damage the bacterial cell membrane by inducing lipid peroxidation and DNA damage.^31^ Furthermore, MOFs may prevent the development of biofilms by killing bacteria or disrupting their quorum sensing, a communication system that regulates their collective behavior.^32^ Examples of antibacterial and antibiofilm MOFs are NH_2_-MIL-125,^33^ ZIF-67,^34^ and Ni-MOF.^35^ MOFs can also act as antioxidants by scavenging FRs, which can cause oxidative stress and inflammation in the body. Some examples of antioxidant MOFs are Ta-MOF,^36^ Al-MOF,^37^ and Mg-gallate-MOF.^38^

Herein, we present two novel MOFs resembling the PCP subclass of MOFs based on zinc or cobalt with 4,6-diamino-2-pyrimidinethiol as an organic linker. They were labeled Zn-MOF and Co-MOF. We investigated their antibacterial, antioxidant, and antibiofilm activities and found them to exhibit high efficacy. The antibacterial activities were assessed *via* the measures of inhibitory zone diameter (IZD), minimum bactericidal concentration (MBC), and minimum inhibitory concentration (MIC). Antibacterial activity was tested against four pathogenic bacteria: clinical isolates of *Staphylococcus aureus* and *Pseudomonas aeruginosa*, as well as their standard strains, *Staphylococcus aureus* ATCC 6538 and *Pseudomonas aeruginosa* ATCC 9027. Additionally, an antibiofilm inhibition assay was conducted for *Staphylococcus aureus* ATCC 6538, and a DPPH scavenging assay was used to illustrate the antioxidant activities of the MOFs. A comprehensive comparison of the characteristic properties of the MOFs was conducted, focusing on antioxidant, antibacterial, and antibiofilm efficacies.

## 2. Experimental section

### 2.1 Chemicals, agars, and antibiotics

All the chemicals, agars, and antibiotics were of analytical grade and were purchased from commercial suppliers, such as Merck, Sigma-Aldrich, Liofilchem, and ThermoFisher Scientific. Glacial acetic acid, methanol, ethanol, dimethylformamide (DMF), dimethyl sulfoxide (DMSO), and 4,6-diamino-2-pyrimidinethiol were obtained from Merck KGaA (Darmstadt, Germany). Ascorbic acid (C_6_H_8_O_6_), phosphate-buffered saline (PBS), cobalt nitrate hexahydrate (Co(NO_3_)_2_·6H_2_O), crystal violet (C_25_N_3_H_30_Cl), and zinc nitrate hexahydrate (Zn(NO_3_)_2_·6H_2_O) were obtained from Sigma-Aldrich (Saint Louis, MO, USA). In the Bioinorganic Lab, double-deionized water (DDW) and sterilized normal saline (NS) were available. Mueller Hinton Agar (MHA), Nutrient Broth (NB), Mannitol Salt Agar (MSA), Blood Agar Base (BAB), and Sorbitol MacConkey Agar (SMAC) were purchased from Liofilchem S.r.l., Italy. Finally, ThermoFisher Scientific in the USA provides standard antibiotics.

### 2.2 Bacteria identification

**Fig. 1:**
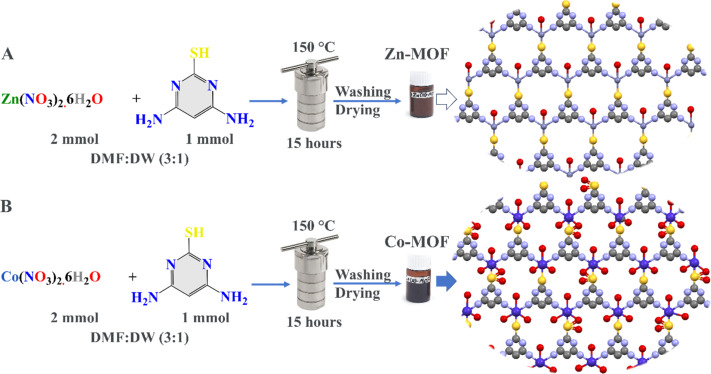
Schematic diagram for the hydrothermal synthesis of (A) Zn-MOFs and (B) Co-MOFs.

Throughout the investigation, bacterial strains, including standard strains (*Staphylococcus aureus* ATCC 6538 and *Pseudomonas aeruginosa* ATCC 9027), and clinical isolates of both species were used. The fully automated VITEK® 2 system (BioMérieux, Marcy-L'Etoile, France) was used to identify them. Following the identification of the species, the Kirby–Bauer test was used to assess the antibiotic susceptibility of each of the following antibiotics, yielding the profiles that are shown in Table 3S and Fig. 1S:† azithromycin (AZM), ceftriaxone (CTR), aztreonam (AT), gentamicin (CN), nalidixic acid (ND), nitrofurantoin (NIT or F), amoxicillin/clavulanic acid (AMC), oxacillin (OX), ampicillin (AMP), rifampicin (RA), clarithromycin (CLR), meropenem (MRP), and erythromycin (E).^39^

### 2.3 Synthesis of Zn and Co MOFs

In this work, Zn-MOF and Co-MOF were prepared in several steps. Initially, solutions containing 4,6-diamino-2-pyrimidinethiol (1 mmol) in 10 mL of a solution mixture of DMF : DW (3 : 1) were prepared separately in two beakers. The solutions were then mixed separately with 2 mmol of Zn(NO_3_)_2_·6H_2_O and Co(NO_3_)_2_·6H_2_O to design Zn-MOF and Co-MOF, respectively. The metal ion mixtures and the organic linker were stirred for 30 minutes. The mixtures were added to 100 mL Teflon-lined autoclaves, sealed, and heated in an oven at 150 °C for 15 hours. After the heating process, the autoclaves were allowed to cool naturally in air, and after the reactions, the powders were obtained. The formed powders were washed several times with 70% ethanol and isolated by centrifugation at 5000 rpm. Finally, the powders were dried and stored in brown bottles for biomedical applications (Fig. 1).

### 2.4 Instrumentation and characterization

An incubator shaker (Innova 43, Eppendorf AG, Germany) was used to promote optimal growth, while an orbital shaker (IKA, model® KS130 basic) was employed to appropriately stain the biomasses. An ELx800 microplate reader from BioTek in the USA was utilized to determine the optical density (OD) of the bacterial growth medium and the percentage inhibition of the biofilm. The chemical composition, magnetic properties, crystalline structure, and morphology of the MOFs were thoroughly examined using various microscopy and spectroscopy methods. Thermal gravimetric analysis (TGA) and differential scanning calorimetry (DSC) were used to analyze the thermal stability, weight losses, and species adsorbed within the MOF using a Setaram DSC131 EVO and PerkinElmer Diamond TGA/DTA. The crystal structure was analyzed using Powder X-Ray Diffraction (PXRD) data with the assistance of Mercury, ChemDraw's 3D visualization, and X'Pert HighScore PRO software for electronic and structural purposes. PXRD patterns were obtained using a monochromatized X-ray beam with Cu Kα radiation at room temperature on a 2*θ* scale ranging from 10° to 80°. The particle size distributions and elemental compositions of the MOFs were assessed using Field Emission Scanning Electron Microscopy (FE-SEM) and energy dispersive X-ray spectroscopy (EDS) with the ZEISS Sigma 300 instrument.

The ThermoFisher Scientific K-Alpha X-ray Photoelectron Spectrometer (XPS) was used to analyze the electronic states and elemental compositions on the surface. Fourier transform infrared (FTIR) spectra with a resolution of 4 cm^−1^ in the 400–4000 cm^−1^ range were recorded using PerkinElmer's instrument (PerkinElmer, Inc., USA). A Cary 60 UV-visible spectrophotometer (Agilent Technologies Inc., USA) was used to produce UV-visible absorption spectra in the 200–800 nm range. Brunauer Emmett Teller (BET) isotherms were used to analyze the surface area, pore volume, and pore diameter of the material using an automated gas sorption analyzer from Quantachrome (USA). The magnetic property and oxidation state of the MOFs were characterized using the magnetic susceptibility balance from Sherwood Scientific Ltd. (MKII, UK). The synthesized MOFs were subjected to an inductively coupled plasma optical emission spectrometer (ICP-OES, PerkinElmer Optima 2100 DV, USA) to determine the concentration of leached cobalt and zinc.

### 2.5 Antibacterial activity

The synthesized MOFs were evaluated owing to their antibacterial properties against Gram-positive (*S. aureus*) and Gram-negative (*P. aeruginosa*) bacteria using guidelines from the Clinical and Laboratory Standards Institute (CLSI, M100, 30th ed. 2020). Bacterial strains were cultured overnight on MHA plates, and a single colony was selected and inoculated into a 1 : 1 mixture of sterilized NS and NB solutions. The bacterial suspension was allowed to grow for two hours, resulting in a concentration of 10^8^ CFU mL^−1^, equivalent to the 0.5 McFarland standard. The IZD was determined by adding 80 μL of MOF suspensions to each well at a concentration of 0.64 mg dL^−1^ after transferring the prepared inoculum to the MHA plates.

**Fig. 2:**
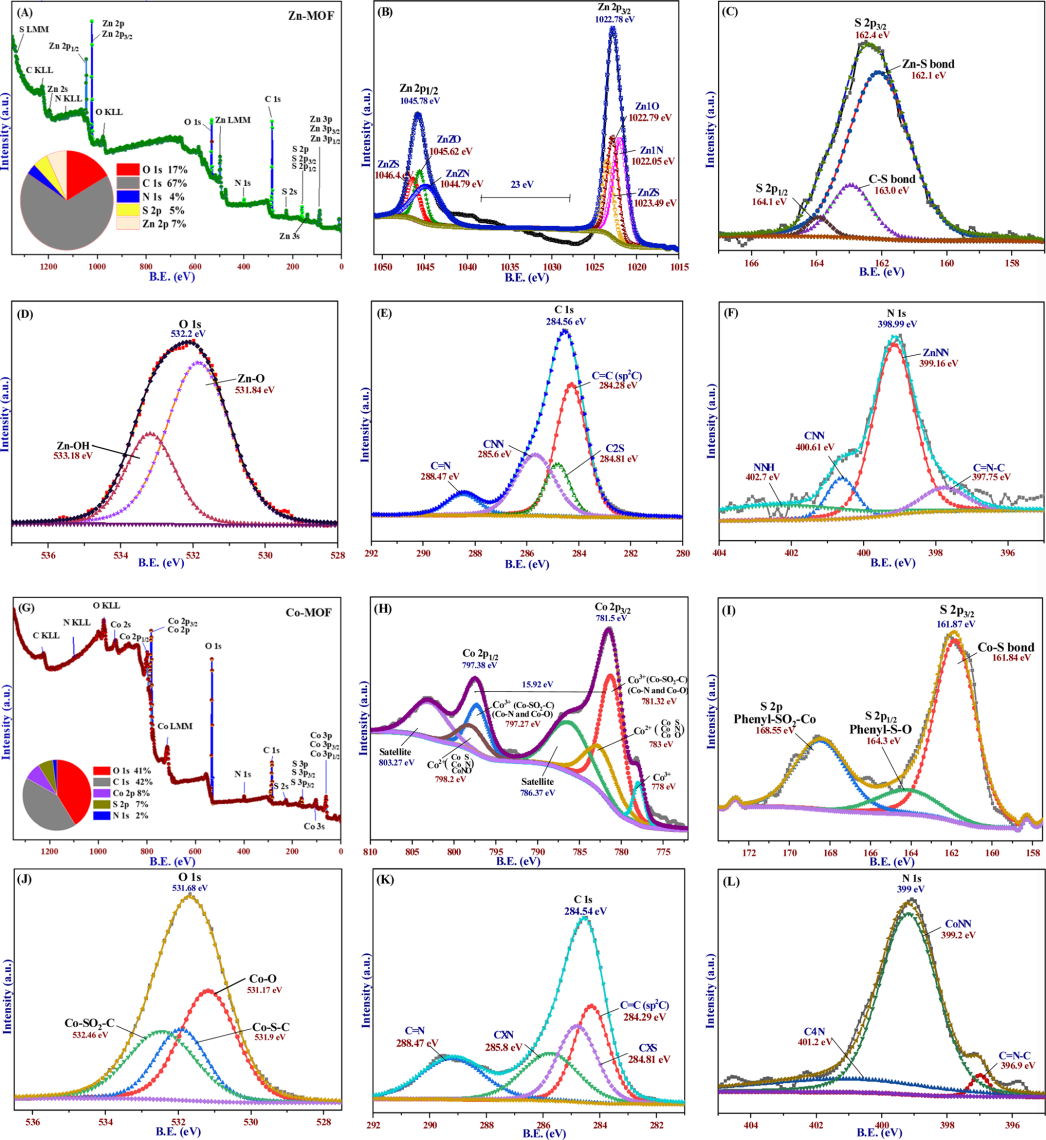
XPS spectra of the Zn-MOF: (A) survey spectrum, (B) Zn 2p, (C) S 2p, (D) O 1s, (E) C 1s, and (F) N 1s. XPS spectra of the Co-MOF: (G) survey spectrum, (H) Co 2p, (I) S 2p, (J) O 1s, (K) C 1s, and (L) N 1s.

The MIC of the MOFs was assessed by treating a series of two-fold concentrations, starting from 10 μg mL^−1^ and ranging up to 2.56 mg mL^−1^, using DMSO as a solvent. The study involved mixing MOFs (0.5 mL), bacterial inoculum (0.5 mL), and NB (1 mL) in sterilized tubes with a positive control group consisting of bacteria and DMSO and a negative control group, including NB and MOFs. Tubes were incubated at 37 °C for 24 hours compared to controls, and their MIC values were confirmed by transferring 300 μL to a sterilized microplate and recording the OD at 600 nm. The positive controls exhibited ODs above 0.09, while the negative controls exhibited an OD of 0.042 or lower, as shown in Tables 4S, 5S and Fig. 2S (ESI†). The MBC was determined by spreading a loopful from the culture wells on the MHA plates after no visible growth was observed and incubating at 37 °C for 24 hours.

### 2.6 Antibiofilm inhibition assay

The assessment of biofilm degradation *via* Zn-MOF and Co-MOF was carried out using the spectrophotometric method against *Staphylococcus aureus* ATCC 6538, following the procedure outlined by Haney *et al.*, 2021, with some modifications.^40^ To provide a brief overview, *S. aureus* bacteria were cultivated on an MHA medium from a cryogenic stock. The cultures were incubated for 24 hours at 37 °C to facilitate the formation of single colonies. The experiment involved preparing overnight cultures in the required tubes, with each tube containing 5 mL of NB medium. The tubes were inoculated with three to five colonies, which were carefully selected based on their similar size and morphology. The bacterial cultures were incubated at 37 °C for 24 hours using an incubator shaker (Innova 43, Eppendorf AG, Germany) to facilitate optimal growth.

For the experimental setup, a sterilized 96-well microtiter plate was utilized. In each well of the plate, the following components were added: 20 μL of bacterial suspensions, 130 μL of NB, and 150 μL of two-fold concentrations of the MOFs, starting from 10 μg mL^−1^ to 5.12 mg mL^−1^. A positive control was designed by combining 20 μL of bacterial suspensions, 130 μL of NB, and 150 μL of DDW, while a negative control was established by mixing 150 μL of NB and 150 μL of DDW. The plate was then gently shaken for 10 minutes and incubated under static conditions at 37 °C for 24 hours.

To conduct the biofilm inhibition assay, the entire medium from the plate was discarded, and the plate was rinsed four times with 350 μL of PBS buffer. It is important to dispose of the non-attached cell waste in containers containing 5% sodium hypochlorite. After rinsing, the plate was inverted on a dry paper towel and lightly tapped to remove any excess water. The attached biomasses in each well were stained by adding 200 μL of crystal violet (0.1%) and incubated using an orbital shaker (IKA, model® KS130 basic) set at 240 rpm for 30 minutes. The stain with excess biomass was discarded from the wells and washed four times with DDW. The plate was allowed to dry at room temperature. The suspended crystal violet (CV) stain was solubilized with 200 μL of a 30% glacial acetic acid solution, and the OD was measured. The measured optical density (OD) values served as a measure of the presence of bacterial biofilm, which was subsequently transformed into a percentage representing the extent of inhibition (Tables 6S and 7S with Fig. 6S†). The OD of the blank was subtracted from all values. The percentage (%) of biofilm inhibition was calculated using the formula described below (eqn (1)). Subsequently, the IC_50_ values of the antibiofilm inhibition of the MOFs were ascertained by employing nonlinear regression of the curve using GraphPad Prism 8 software (San Diego, CA, USA, 2021).1
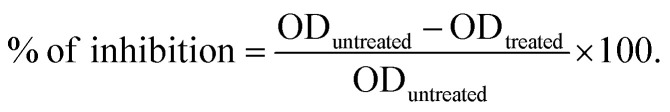


### 2.7 DPPH^.^ Radical scavenging assay

The radical scavenging activity test of 2,2-diphenyl-1-picrylhydrazyl (DPPH) was conducted using spectroscopic techniques, following a standard procedure with some modifications based on reported methods.^41^ A DPPH solution of 0.1 mM was prepared in 100 mL of methanol. For the assay, a 1 mL solution of 0.1 mM DPPH was used. To this DPPH solution, 1 mL of MOFs was added. The MOFs were prepared as a two-fold serial solution, ranging from 10 μg mL^−1^ to 2.56 mg mL^−1^ in DMSO.

The reduction in DPPH was observed by reading absorbance at 517 nm after incubating the tube on an orbital shaker in the dark at room temperature for about 30 minutes. As a control, the absorbance of 1 mL DPPH with 1 mL DMSO was measured, and as a standard, the absorbance of 1 mL DPPH with 1 mL of a two-fold series concentration of ascorbic acid (AA) ranging from 10 μg mL^−1^ to 256 μg mL^−1^ was recorded at 517 nm. For all samples, the percentage inhibition was calculated using the following equation (eqn (2)):2
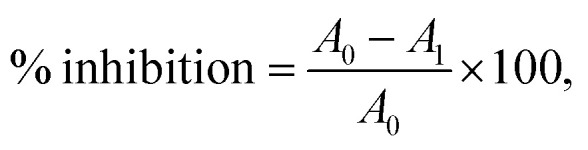
where *A*_0_ represents the absorbance of the control (DPPH + DMSO) and *A*_1_ represents the absorbance of the sample (DPPH + samples). We determined the concentration required to scavenge 50% of the DPPH radical (IC_50_ value). To achieve this, we plotted a graph correlating the percentage inhibition with the concentration of the MOFs. The IC_50_ values of the MOFs were subsequently determined using nonlinear regression analysis *via* GraphPad Prism 8 software. The IC_50_ value is commonly employed to compare the antioxidant activities of different samples.

## 3. Results and discussion

### 3.1 Characterization of the MOFs

X-ray photoelectron spectroscopy (XPS) was utilized to study the chemical compositions, oxidation states, and surface functionalities of the MOFs, as depicted in Fig. 2. Survey spectrum (Fig. 2A) results revealed that the surface of Zn-MOF primarily consisted of oxygen (17%), carbon (67%), zinc (7%), sulfur (5%), and nitrogen (4%). The high-resolution spectra of Zn-MOF (Fig. 2B–F) provided further evidence for understanding the structural features and oxidation state of the elements within the Zn-MOF. Two symmetrical characteristic peaks were specifically observed in the Zn 2p spectra at binding energies of 1022.78 eV and 1045.78 eV. These peaks, with a spin–orbit splitting of 23 eV, were identified as Zn 2p_3/2_ and Zn 2p_1/2_, respectively.^42^ Notably, these peaks aligned well with the reported binding energy for peak separation by other researchers, and there was no evidence of zinc oxide in the Zn-MOF.^42^ Additionally, these peaks represented distinct components of different functional groups, such as Zn–N, Zn–O, and Zn–S, on the surface of the molecule.^43^ The XPS signal of S 2p showed a peak at 162.4 eV for S 2p_3/2_, which could be attributed to S–Zn at 162.1 eV and S–C at 163.1 eV, along with a peak at 164.1 eV for S 2p_1/2_.^44^ The O 1s XPS spectrum showed two discrete peaks at 532.2 eV (Fig. 2D). One of the peaks at 531.84 eV arose from the coordination of water molecules with a Zn-cluster-based MOF. Once the water molecules on the surface of the Zn-MOF deprotonated, the peak at 533.18 eV matched Zn–OH.^45^ The C 1s spectrum (Fig. 2E) displayed four distinct peaks corresponding to various carbon bonds involving sp^2^ and sp^3^ hybridization.^46^ These peaks corresponded to C=C, C–S, C–N, and C=N, and their binding energies were 284.28, 284.81, 285.6, and 288.47 eV, respectively.

The N 1s spectrum, as depicted in Fig. 2F, showed a single low-intensity peak with the presence of shoulders at a binding energy of 398.99 eV. The peak could be attributed to four different environments of nitrogen bonds: C=N–C, Zn–N, C–N, and N–H; they were identified at 397.75, 399.16, 400.61, and 402.7 eV, respectively.^42^ According to the survey spectrum of the Co-MOF (Fig. 2G), the primary elements found on the surface of the MOF were carbon (42%), oxygen (41%), cobalt (8%), sulfur (7%), and nitrogen (2%). The findings shown in Fig. 2H demonstrated that the Co 2p spectrum of the Co-MOF was deconvoluted into two distinct peaks consistent with Co 2p_3/2_ and Co 2p_1/2_, which had binding energies of 781.5 and 797.38 eV, respectively. Moreover, the presence of Co^3+^ along with Co^2+^ was further supported by two minor additional peaks at 786.37 and 798.2 eV, which corresponded to the satellite peaks of Co^3+^.^47^ The characteristics and location of these peaks indicate the oxidation–reduction of Co atoms. The intensity of the Co 2p peak is directly linked to the cobalt content in the Co-MOF.

To examine the oxidation state of the cobalt species, Co 2p_3/2_ was deconvoluted into three peaks. Two of the peaks corresponded to the different environments of Co^3+^ species at binding energies of 778 and 781.32 eV, while the third peak at the binding energy of 783 eV was attributed to Co^2+^. This phenomenon was further supported by the magnetic behavior of the Co-MOF. However, the presence of oxidative sulfur (Co–O–SO) on the surface of the Co-MOF is confirmed by two asymmetrical peaks at 161.87 and 168.55 eV (Fig. 2I). These peaks also indicate the absence of free elemental sulfur.^44^ The peak at 168.55 eV is explained by Co–S–O, while the peak at 161.87 eV can be deconvoluted into two peaks at 161.84 and 164.3 eV, which correspond to the Co–S bond for S 2p_3/2_ and the phenyl–S–O bond for S 2p_1/2_, respectively.^44^ In the O 1s spectrum (Fig. 2J), three distinct peaks were observed at energy values of 531.17, 531.9, and 532.46 eV. These peaks correspond to different chemical bonds involving oxygen atoms on the surface of the structure. The peaks are associated with Co–O, Co–S–C, and Co–SO_2_–C bonds, respectively.^48^ The C 1s spectrum with high resolution had four peaks at 284.29, 284.81, 285.8, and 288.47 eV, as shown in Fig. 2K. They were associated with C=C, C–S, C–N, and C=N bonds, respectively. The Co-MOF N 1s XPS spectrum (Fig. 2L) had one peak at 399 eV and a weak shoulder at 396.9 eV, which were related to C=N–C for the imine part of the organic linker. The main N 1s peak could be split into two peaks at 399.2 and 401.2 eV binding energies, which were related to the Co–N and C–N bonds.

**Fig. 3:**
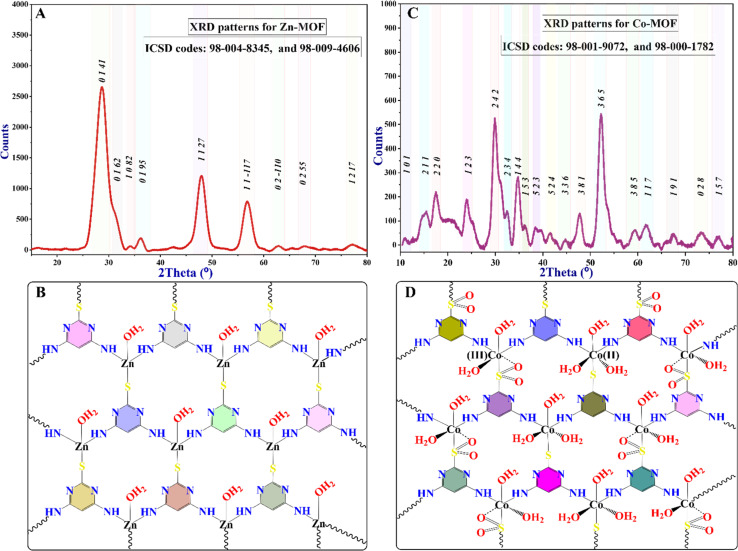
(A) XRD spectra of the Zn-MOF, which are compared and matched with the following ICSD numbers: 98-004-8345 and 98-009-4606. (B) Simulated structure of the Zn-MOF. (C) XRD spectra of the Co-MOF are consistent with the following ICSD numbers: 98-001-9072 and 98-000-1782. (D) Simulated structure of the Co-MOF.

The X-ray powder diffraction (XRD) data of the synthesized MOFs confirm their crystalline properties and reveal different structural analogs, as indicated by the various characteristic peaks shown in Fig. 3. Diffraction peaks at 28.59°, 31.60°, 34.35°, 36.17°, 47.69°, 56.63°, 62.76°, 67.87°, and 77.15° were observed, corresponding to the (0141), (0162), (1082), (0195), (1127), (11−117), (02−110), (0255), and (1217) planes of Zn-MOF, respectively. The Debye–Scherrer equation was used to calculate the crystallite sizes of 31 nm for Zn-MOF. Additionally, most of the peaks indicated the formation of a tetrahedral geometrical structure, as confirmed by the XPS, UV-visible, and magnetic properties of the Zn-MOF. The PXRD peaks were aligned with ICSD files 98-004-8345 and 98-009-4606, as alternatives to the standard JCPDS file.

The diffraction peak positions of the Co-MOF, as shown in Fig. 3C, confirm its high purity and crystallinity. This observation indicates the formation of an octahedral geometry. As shown in Fig. 3, zinc ions in Zn-MOF have a tetrahedral coordination geometry with a coordination number of four due to their smaller size, forming stable complexes with sp^3^ hybridization. Cobalt ions, with their larger ionic radius and additional d-orbitals, adopt an octahedral coordination geometry, accommodating six ligands with sp^3^d^3^ hybridization in Co-MOF. This difference in coordination numbers affects interactions with ligands. Various characteristic peaks observed in the PXRD pattern corroborate this finding. Additionally, confirmed characteristic data from XPS, UV-visible, and magnetic properties of the Co-MOF further support its crystalline structure.^49^ The Co-MOF exhibits significant diffraction peaks at the following specific angles: 10.68°, 15.27°, 17.37°, 23.9°, 29.78°, 32.94°, 34.73°, 36.60°, 38.38°, 41.63°, 44.70°, 47.76°, 52.43°, 59.42°, 61.79°, 67.69°, 73.46°, and 76.89°. These peaks can be indexed to different (*hkl*) crystallographic planes, specifically (101), (211), (220), (123), (242), (234), (144), (153), (523), (524), (336), (381), (365), (385), (117), (191), (028), and (157), respectively. The peak positions align with the ICSD cards 98-001-9072 and 98-000-1782. The crystallite size of the Co-MOF, determined through PXRD analysis, is approximately 43 nm. The average particle size of MOFs, as observed in the FE-SEM micrographs, is consistent with the crystallite sizes observed in the XRD.

**Fig. 4:**
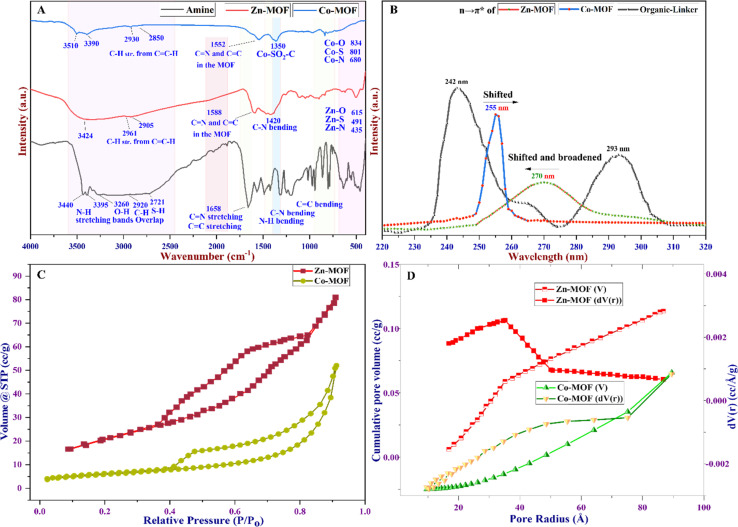
(A) FTIR spectra of Zn- and Co-MOFs, (B) UV-visible spectra of the MOFs, (C) N_2_ adsorption–desorption isotherm, and (D) BJH pore size distribution curve for the MOFs.

Fourier transform infrared spectroscopy (FTIR) characterization spectra are presented in Fig. 4A. The results demonstrate characteristic peaks indicating the formation of MOFs and the disappearance of bands associated with organic linkers. These changes occur due to the bonding between the donor atoms of the ligand and the metal ions; consequently, MOFs are formed. Prior to the reaction, the absorption bands of the organic linker were detected, ranging from 2700 to 3500 cm^−1^. These bands represent the stretching of the C–H, S–H, and N–H bands associated with the free NH_2_ groups.^50^ The stretching vibrations of water molecules adsorbed on the surface of the ligand were also correlated with these findings. However, following the formation of Zn-MOF and Co-MOF, changes were observed in the absorption bands related to the stretching vibration of the N–H bonds.^49^ These bands either shifted or disappeared due to the coordination and bonding between the metal ions and the organic linker within the MOF structure. The absorption bands observed in the range of 2900–3450 cm^−1^ in the Zn-MOF spectra provide evidence of water molecule coordination to the Zn(ii) ion and the consequent disappearance of the N–H bond due to nitrogen coordination with Zn ions.^51^ Moreover, the absorption peaks detected between 1650 and 1400 cm^−1^ clearly indicate the presence of C–H bending vibrations and validate the absence of the C–SO_2_–Zn bond, as corroborated by XPS and XRD characteristic data.

Specifically, the absorption bands at 1588 cm^−1^ in the Zn-MOF and at 1552 cm^−1^ in the Co-MOF signify the bending vibration of the C=C and C=N bonds, respectively. Additionally, the absorption peak observed at 1420 cm^−1^ suggests the bending vibrations of N–H molecules. Notably, in the Co-MOF, the characteristic bands observed at 1350 cm^−1^ confirm the presence of Co–SO_2_–C bonds. These may arise from the oxidation–reduction process of Co ions within the MOF centers, leading to the oxidation of sulfur in half of the MOF structural molecule.^52^ The Zn-MOF and Co-MOF demonstrate significant M–O stretching bands, resulting from the asymmetric and symmetric vibrations of water molecules bonded to the metal ions. These vibrations cause slight frequency shifts owing to conjugation with the carbon–nitrogen bond. Additionally, low-intensity bending vibrations are observed at 615, 491, and 435 cm^−1^ in the Zn-MOF, corresponding to M–O, M–S, and M–O bonds, respectively. Similarly, in the Co-MOF, bands at 834, 801, and 680 cm^−1^ are observed, corresponding to M–O, M–S, and M–O bonds, respectively.^53^

UV-visible techniques are widely employed for the characterization of Zn- and Co-MOFs, and they play a crucial role in elucidating the structural and optical properties of the compounds. One key aspect observed in the UV-visible spectra, as depicted in Fig. 4B, is the difference between the UV peaks of the linker (amine) and those of the MOFs formed. The ligand exhibits two peaks at 243 and 293 nm. In contrast, the UV peak of the formed Zn-MOF at 270 nm is shifted and broadened to a shorter wavelength compared to that of the ligand, while the UV peak of the Co-MOF at 255 nm is shifted and narrowed to a longer wavelength compared to the ligand peaks. This discrepancy arises from several factors. First, the presence of metal ions within the MOF structure introduces new electronic states that can interact with the ligand, leading to changes in the energy levels of the resulting n–π* transitions. Additionally, the arrangement and coordination of the ligand with the metal ions in the MOF framework can influence conjugation and electronic delocalization, thereby causing a shift in the UV peak of the MOFs compared to the ligand. This shift can provide valuable insights into the structural changes that occur upon Zn- and Co-MOF formation. This can indicate the ligand–metal ion interaction differently, the formation of new metal–ligand bonds, and the alteration of the overall structural integrity of both MOFs.

The pore diameter, pore volume, and specific surface area were determined using Brunauer–Emmett–Teller (BET), Barrett–Joyner–Halenda (BJH), and Langmuir methods, analyzing the nitrogen adsorption–desorption isotherm at a constant temperature. According to the BET isotherm, the Zn-MOF exhibited a specific surface area of 45.88 m^2^ g^−1^. Conversely, the Co-MOF exhibited a surface area of 20.39 m^2^ g^−1^, as shown in Table 1 and Fig. 4C. The reduction in the Co-MOF specific surface area compared to the Zn-MOF may be attributed to the existence of substantial mesoporous interlayer voids inside the Zn-MOF structure. The use of the BJH isotherm and the pore size distribution curve shown in Fig. 4D confirm the existence of mesoporous properties in the MOFs.^54^ These features primarily originate from the high number of voids in the Co-MOF compared to the Zn-MOF. It was observed that Co-MOF had comparable total pore volumes, which were greater than the pore volume of Zn-MOF, as indicated in Table 1. The results displayed in Fig. 4D and Table 1 demonstrate that the pore diameter of Co-MOF obtained was 21.65 nm compared to the 7.02 nm diameter of Zn-MOF, confirming the presence of mesoporosity in the MOF structures.^29^ Among the six types of BET isotherms, the BET isotherm of the MOFs confirms hysteresis in a specific type known as the type IV BET isotherm, as depicted in Fig. 4C.^55^ The type IV isotherm exhibits two distinct patterns, both associated with pore width. When the width exceeds a critical threshold determined by the adsorption properties and temperature of the material, this type of isotherm can be obtained at high relative pressure in the pores.

**Table 1:** A brief overview of the N_2_ adsorption–desorption isotherm study using the BET, BJH, and Langmuir techniques

Physisorption characteristic data		Zn-MOF	Co-MOF
BET summary	Specific surface area (m^2^ g^−1^)	45.883	20.393
BJH adsorption and desorption summary	Surface area (m^2^ g^−1^)	59.136	23.698
	Pore volume (cm^3^ g^−1^)	0.114	0.081
	Pore radius d*V*(*r*) (Å)	35.149	108.263
	Pore diameter (nm)	7.03	21.65
Langmuir summary	Surface area (m^2^ g^−1^)	787.399	363.132

This observation could be attributed to the various structural analogs of MOFs, including factors such as the connectivity between ligands and metals, as well as the distribution of pore sizes throughout the structure and the geometry of the pores. The high surface area and large pore diameter typically correspond to a greater number of active sites, leading to higher catalytic activity.^54^ Consequently, Zn-MOF displays a substantial surface area due to the greater quantity of active sites in comparison to Co-MOF, but Co-MOF has a large pore diameter. The Langmuir isotherm analysis revealed that Zn-MOF exhibited a monolayer surface area of 787.39 m^2^ g^−1^, while Co-MOF exhibited a surface area of 363.13 m^2^ g^−1^. The Langmuir isotherm assumes a monolayer adsorption process, enabling the determination of essential parameters, such as maximum adsorption capacity and equilibrium constant. These parameters offer valuable insights into the adsorption phenomenon and surface interactions during catalytic processes.

**Fig. 5:**
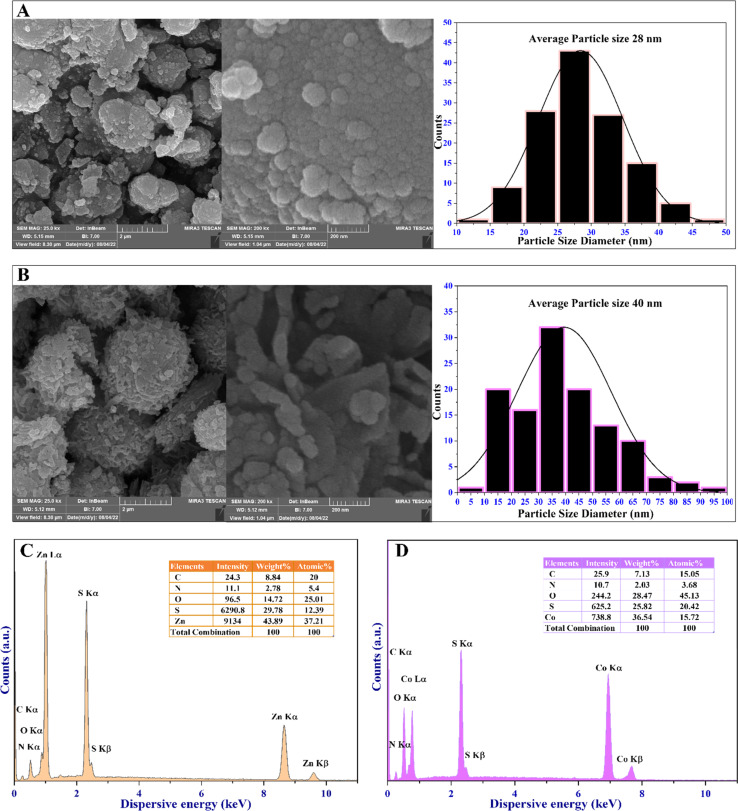
FESEM-EDX analysis of (A) the Zn-MOF and (B) Co-MOF. FESEM micrographs showing the morphology of the Zn-MOF and Co-MOF with particle size distribution histograms. (C) EDX spectra of the Zn-MOF, and (D) EDX spectra of the Co-MOF.

High-resolution field-emission scanning electron microscopy (FESEM) was utilized to evaluate the surface morphology, particle size distribution, and structural features of the MOFs. This characterization technique provides valuable insights into the microstructure and surface features of MOFs. The obtained FESEM micrograph, the particle size distribution histograms, and the EDX spectra of the MOFs are depicted in Fig. 5. They show a spherical morphology and varying degrees of agglomeration, with grains combined. The FESEM approach, coupled with EDX, is used to precisely identify the chemical composition of the synthesized MOFs (Fig. 5). Both MOFs display differences in the rate of aggregation, morphological configuration, and distribution of particle sizes. However, the presence of zinc and cobalt metal ions within the 4,6-diamino-2-pyrimidinethiol framework, acting as an organic linker, is evidenced by the uniform distribution of Zn, Co, C, N, and S elements. Additionally, O represents coordinated water molecules, as shown in Fig. 5C and D.

Moreover, EDX mapping images, along with XPS analysis (Fig. 2), demonstrate a consistent distribution of Zn and Co throughout the MOF materials. This incorporation of metal species with oxygen, nitrogen, and sulfur groups facilitates the formation of various voids as active sites for the catalytic activity of these materials. Consequently, a well-defined active site forms on the surface of the MOFs, thereby enhancing their antibacterial, antibiofilm, and antioxidant capacities.

The FESEM micrograph offered valuable insights into the particle properties of the MOFs. Zn-MOF displayed spherical particles of around 28 nm in diameter, while Co-MOF showed semi-spherical particles with an average diameter of 40 nm. The EDX elemental mappings delineate the elemental composition and distribution of the MOFs, confirming the presence of major and trace elements, revealing spatial distribution, and providing semi-quantitative information within the EDX mapping. Additionally, it generates elemental mapping images, reveals interactions between elements, and can detect surface contaminants. The EDX elemental mapping of the MOFs confirms the formation of the MOFs with all elements within the MOF structure, as shown in the ESI in Fig. 7S and 8S.† The FESEM and EDX mapping images conclude that the weak repulsion caused by the diamagnetic characteristic of the MOFs inhibits the formation of larger agglomerations and grain combinations in the MOFs.

The diamagnetic effect creates a weak repulsion among the MOFs, which reduces the tendency for agglomeration.^56^ Moreover, the type of metal ions and the shape of the MOFs affect their surface features, particle sizes, and clustering behavior. These factors explain why Zn-MOF and Co-MOF have different patterns of agglomeration and grain formation.

**Fig. 6:**
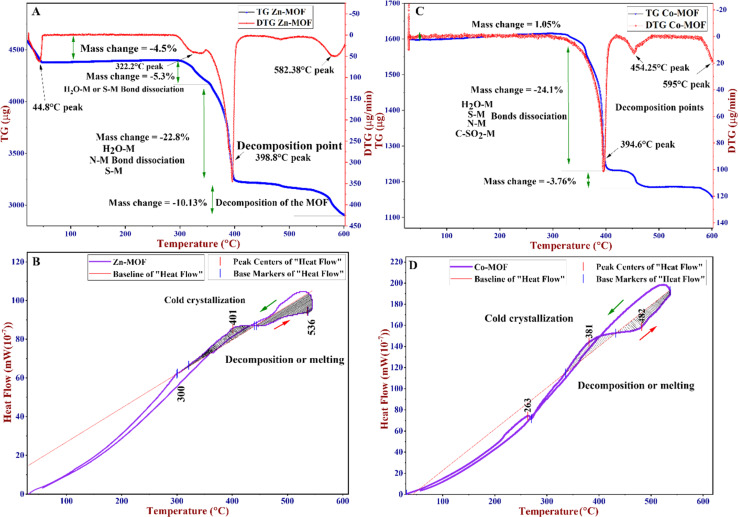
(A) TG/DTG of the Zn-MOF, (B) DSC of the Zn-MOF, (C) TG/DTG of the Co-MOF, and (D) DSC of the Co-MOF.

Differential Thermogravimetry (DTG), Differential Scanning Calorimetry (DSC), and Thermogravimetric Analysis (TGA) were used to evaluate the thermal stability of the MOFs. The experiments were carried out at temperatures ranging from 30 to 600 °C, with a heating rate of 10 °C min^−1^. These techniques are commonly used to understand the thermal behavior of MOFs. The thermograms are depicted in Fig. 6. The TGA/DTA curves were used to analyze the decomposition steps of the MOFs. TGA can be utilized to differentiate between water molecules situated and coordinated in the lattice structure of the MOFs or adsorbed on the surface of the MOFs in terms of coordinated water molecules in the structure of the MOFs.^57^ The elimination of water molecules attached to the surface of MOFs usually occurs at temperatures below 200 °C, corresponding to the water molecules linked to the surface of the MOFs.

However, water molecules coordinated in the lattice structure are usually expelled above 200 °C. As shown in Fig. 6A, a weight loss of 4.5% around 45 °C could be attributed to the loss of adsorbed water molecules on the surface of the Zn-MOF, but this was not observed in the Co-MOF (Fig. 6C). This behavior could be attributed to the stability of water molecules related to Zn-MOF, which is strongly related to the organic linker and metal ions and is not observed in Co-MOF.^58^ The TGA of both MOFs reveals a continuous reduction in weight starting at around 290 °C and lasting until approximately 400 °C.

**Fig. 7:**
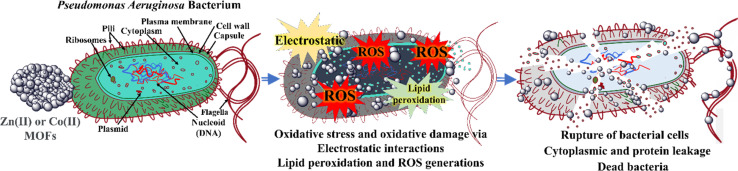
Proposed mechanism of the antibacterial activities of the Zn-MOF and Co-MOF on the clinical isolate of *Pseudomonas aeruginosa* bacterium.^31^

Based on the DTG, the Zn-MOF was decomposed at 398.8 °C and the Co-MOF was decomposed at 394.6 °C. At these points, the MOF structures underwent decomposition, as further confirmed by DSC thermograms. Initially, during the decomposition process, adsorbed water, along with the dehydration of coordinated water molecules, deamination, and dethiolation, occurred. Furthermore, the TGA and DTG curves indicate that the crystallinity of the MOF is completely decomposed at temperatures higher than 500 °C, which is further supported by the DSC curves (Fig. 6B and 7D). DSC is a technique that measures the heat flow of a sample as its temperature changes. It can provide information about various thermal properties of a material, such as heat capacity, phase transitions, crystallization, melting point, and decomposition point. The DSC thermograms of Zn-MOF and Co-MOF showed that phase transition onset occurred at 300 °C and 265 °C, respectively. The exothermic peaks at 401 °C for Zn-MOF and 381 °C for Co-MOF suggested the crystallization of the MOFs. The decomposition of the MOFs was indicated by the endothermic peaks corresponding to heat absorption during the phase transition at 536 °C for Zn-MOF and 482 °C for Co-MOF. These results confirmed that Zn-MOF had higher thermal stability than Co-MOF. The magnetic susceptibility balance from Sherwood Scientific Ltd. (MKII, UK) was employed to determine the oxidation state and magnetic properties of the MOFs. The results agreed with the XPS analysis techniques applied to the MOFs. The mass susceptibility (*χ*_m_)/volume susceptibility (*χ*_v_) of the MOFs were used to measure the magnetic properties of Zn-MOF and Co-MOF. The results indicated that Co-MOF displayed diamagnetic characteristics, with most of the Co(ii) in its MOF structure oxidized to Co(iii), as corroborated by the XPS data. The XPS data revealed that the Co-MOF structure contained Co^2+^ and Co^3+^ sites (Fig. 2H). Conversely, Zn(ii) in Zn-MOF remained in its oxidation state with diamagnetic behavior. Based on the characterization techniques, Zn in Zn-MOF exhibited a tetrahedral structure with the hybridization of sp^3^, while Co-MOF possessed an octahedral structure with the hybridization of d^2^sp^3^.

### 3.2 Antibacterial activity of the MOFs

**Fig. 8:**
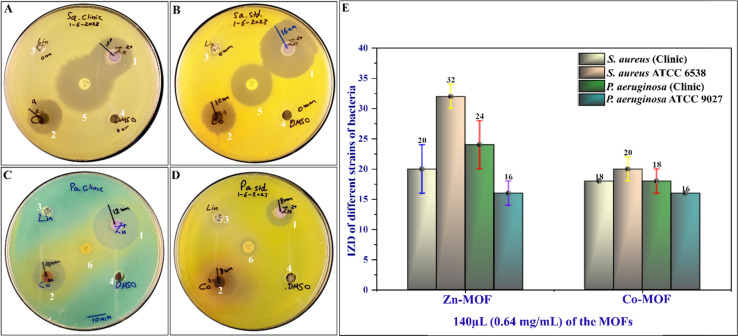
Antimicrobial activity against clinical isolates of (A) *Staphylococcus aureus* and (C) *Pseudomonas aeruginosa*, as well as their standard strains (B) *Staphylococcus aureus* ATCC 653 and (D) *Pseudomonas aeruginosa* ATCC 9027. (E) A bar graph with error bars illustrating the analysis of antimicrobial activity data. The samples subjected to testing included (1) the Zn-MOF, (2) the Co-MOF, (3) organic linker, (4) DMSO, (5) ceftriaxone (CTR), and (6) clarithromycin (CLR).

The antibacterial activity of the synthesized MOFs was evaluated against standard strains and clinical isolates of both Gram-positive and Gram-negative bacteria. The evaluation was conducted in duplicate. The results demonstrated that both Zn-MOF and Co-MOF exhibited significant antibacterial activity against all tested bacteria, with Zn-MOF being more effective than Co-MOF based on the IZD, as shown in Fig. 8. The MIC and MBC of the MOFs are presented in Table 2. Notably, both MOFs exhibited nearly the same potent antibacterial activity. The proposed mechanism underlying the antibacterial activity of the MOFs is depicted in Fig. 7. These MOFs induce bacterial cell death through various detrimental effects, including the generation of ROS, electrostatic interaction, and lipid peroxidation.^31,34^ These effects occur when the MOFs bind to the bacteria, causing oxidative damage. Ultimately, this severe damage disrupts bacterial cell walls and membranes, leading to cell lysis and death.^31^ When the WDA protocol was applied to evaluate the antibacterial activity of Zn-MOF and Co-MOF, the results indicated that Zn-MOF exhibited superior antibacterial performance compared to Co-MOF. This superiority was evidenced by the larger IZD values obtained for Zn-MOF (as shown in Table 2 and Fig. 8). The observed behavior may be attributed to the smaller particle size and crystallite size of Zn-MOF, as well as its larger surface-to-volume ratio. These factors contribute to the enhanced antibacterial effectiveness of Zn-MOF by enhancing permeability in comparison to Co-MOF.

**Table 2:** Antibacterial activity of the Zn-MOF and Co-MOF using WDA

Bacterial strains	IZD at a concentration of 0.64 mg dL^−1^ (with 80 μL per well)			
	Zn-MOF	Co-MOF	Linker (amine)	DMSO
*S. aureus* (clinic)	20 ± 4 (24) mm	18 ± 0 (18) mm	0 mm	0 mm
*S. aureus* ATCC 6538	32 ± 2 (30) mm	20 ± 2 (22) mm	0 mm	0 mm
*P. aeruginosa* (clinic)	24 ± 4 (20) mm	18 ± 2 (16) mm	0 mm	0 mm
*P. aeruginosa* ATCC 9027	16 ± 2 (18) mm	16 ± 0 (16) mm	0 mm	0 mm

The antimicrobial effectiveness of MOFs and other antimicrobial agents depends heavily on the structures, compositions, surface functionalities, and bacterial cell wall properties of the material. Research has revealed that Gram-positive bacteria, with their thick peptidoglycan layer, encounter difficulties penetrating the surface functionality of MOFs. Conversely, Gram-negative bacteria, possessing a thinner peptidoglycan layer, demonstrate higher sensitivity, resulting in lower MIC values.^59^ For instance, as illustrated in Table 3, both Gram-positive bacteria, such as the clinical isolate of *S. aureus* and its standard strain, *S. aureus* ATCC 6538, exhibit high MIC values of 0.64 mg mL^−1^. In contrast, the Gram-negative bacteria *P. aeruginosa*, both as a clinical isolate and its standard strain, *P. aeruginosa* ATCC 9027, display lower MIC values of 0.08 mg mL^−1^.

**Table 3:** MIC and BMC values of the MOFs

Bacterial strains	Antibacterial activity *via* MICs		Antibacterial activity *via* MBCs	
	Zn-MOF	Co-MOF	Zn-MOF	Co-MOF
*S. aureus* (clinic)	0.64 mg mL^−1^	0.64 mg mL^−1^	2.56 mg mL^−1^	2.56 mg mL^−1^
*S. aureus* ATCC 6538	0.64 mg mL^−1^	0.64 mg mL^−1^	2.56 mg mL^−1^	1.26 mg mL^−1^
*P. aeruginosa* (clinic)	0.08 mg mL^−1^	0.08 mg mL^−1^	2.56 mg mL^−1^	2.56 mg mL^−1^
*P. aeruginosa* ATCC 9027	0.08 mg mL^−1^	0.08 mg mL^−1^	>2.56 mg mL^−1^	>2.56 mg mL^−1^

The cytotoxicity of Zn-MOF and Co-MOF is possibly associated with the production of ROS. These ROS selectively target bacteria while leaving non-bacterial cells unharmed, resulting in damage to the bacterial cell wall and nucleic acids. Such damage can lead to protein leakage and, ultimately, bacterial death. Additionally, the cytotoxic effects of MOFs on bacteria are attributed to their electrostatic interactions.

Both MOFs have different metal nodes and share the same organic linkers. They provide various structural surface charges and functionalities. However, bacteria exhibit various membrane structures that display a negative charge for both Gram-negative and Gram-positive bacteria. This charge arises from their main chemical constituents: teichoic or lipoteichoic acid-containing peptidoglycan in Gram-positive bacteria and phospholipids in Gram-negative bacteria.^60^ XPS and IR techniques partially reveal the presence of positively charged metal ions, such as Co^2+^/Co^3+^ and Zn^2+^, on the surface structure of MOFs.^61,62^ In contrast to bacteria, the surface structure of MOFs is predominantly positive, facilitating interaction with the negatively charged bacterial surface. This interaction induces an electrostatic attraction force,^61^ which may inhibit cell wall synthesis, retard protein synthesis, and interact with bacterial genetic material (DNA), ultimately leading to the destruction of bacterial cell walls and causing protein and cytoplasm leakage, as depicted in Fig. 7.

Both MOFs showed similar MIC values against various bacterial strains, with *S. aureus* strains showing 0.64 mg mL^−1^ and *P. aeruginosa* strains showing 0.08 mg mL^−1^ (as shown in Table 3). The MBC values of the MOFs are also presented in Table 3, indicating that the MOFs exhibited bactericidal activities against *S. aureus* strains, with the MBC values being four times higher than the MIC values (MBC/MIC ≤ 4).^63^ However, when the MOFs were used against the *P. aeruginosa* strains, the MBC values were thirty-two times larger than the MIC values (MBC/MIC > 4).^63^ According to the IZD and MIC results, the MOFs exhibited potent antibacterial activity against all bacteria. Additionally, the MOFs demonstrated bactericidal activity against *S. aureus* strains, while they displayed bacteriostatic activity against *P. aeruginosa* strains based on MBC/MIC ratios. The MBC values of Zn-MOF and Co-MOF against *S. aureus* ATCC 6538 differ, as shown in Table 3. Several factors supported by characterization techniques can be responsible for this variance. The distinct metal oxidation states of Co^II^/Co^III^ and the geometric differences between the MOFs play significant roles, contributing to a synergistic effect that explains this variation. XPS, PXRD, and FTIR corroborate this explanation, suggesting heightened oxidative potential and improved interaction properties, which result in high oxidative stress.^64^ The coordination geometry of the ligands around the metal ion can also have a big effect on how it interacts with bacterial cells, which could change the MBC. MOFs of varying shapes might better reach bacterial membranes or cellular targets, enhancing their effectiveness against bacteria. Notably, functional groups in Co-MOF, such as (–C=C–SO_2_–Co–), may directly interact with bacterial cells or facilitate cellular uptake, potentially enhancing bactericidal efficacy compared to Zn-MOF.

### 3.3 Antibiofilm activity of the MOFs

The antibiofilm activity of Zn-MOF and Co-MOF was investigated to assess their effects on biofilm formation, biofilm disruption, or their ability to remove biofilms formed by *Staphylococcus aureus* ATCC 6538. The ability of *S. aureus* to create biofilms is one of the crucial factors contributing to its virulence, enabling it to resist antibiotics and evade the defense mechanisms of the host. Biofilms are groupings of bacteria or other microbes that attach to surfaces and create a defensive structure composed of extracellular polymeric substances (EPS). These biofilms are highly resistant to antibiotics and the immune response of the host, making them a significant challenge in medical, industrial, and environmental settings. Nowadays, the development of efficient antibacterial and antibiofilm agents against *S. aureus* strains has proven to be quite challenging. The National Institute of Health's epidemiological survey indicates that over 60% of infections stem from microorganisms capable of forming biofilms. Notably, among these microorganisms, *S. aureus* and *P. aeruginosa* are two prominent examples.^65^ Our focus was to explore the impact of Zn-MOF and Co-MOF on the established biofilm of *S. aureus* ATCC 6538 using microtiter plate assays with a CV staining protocol to quantify biofilm biomass.

It is widely accepted that the formation of biofilm biomass is directly linked to the amount of CV adsorbed, which occurs due to interactions with negatively charged bacteria and polysaccharides within the EPS. However, the types of solvents, broth media, and surface types can significantly affect the ability of bacteria to form a biofilm and adhere to a surface. Consequently, the use of DMSO solvent, either alone or in combination with DDW (in a 1 : 5 ratio), affects biofilm formation. Therefore, DDW was used solely to prepare a two-fold serial concentration, which optimally does not affect biofilm growth. Interestingly, these MOFs effectively inhibited the formation of *S. aureus* ATCC 6538 biofilms, as demonstrated by measuring the half maximal inhibitory concentration (IC_50_) for the percentage of biofilm inhibition of these MOFs.

**Fig. 9:**
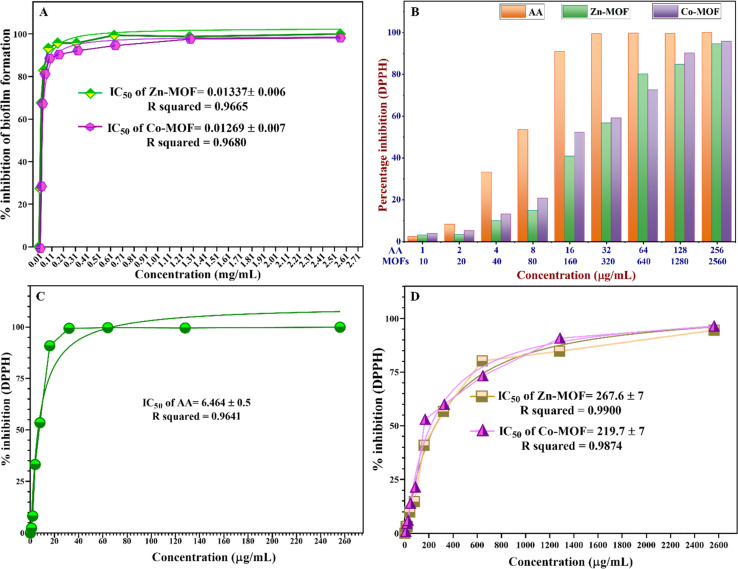
(A) Percentage of biofilm inhibition with a nonlinear regression fitting curve to find IC_50_ values for the MOFs, (B) histograms showing the comparison of the percentage inhibition of DPPH˙ as a function of a two-fold series concentration of the MOFs compared with the standard (AA), (C) nonlinear regression fitting curve of AA to calculate IC_50_, and (D) nonlinear regression curve for antioxidant scavenging activity of MOFs against DPPH.

The IC_50_ value represents the concentration of the MOFs required to inhibit biofilm formation or reduce biofilm biomass by 50%. According to the findings presented in Fig. 9A, the Co-MOF demonstrated a lower IC_50_ value compared to the Zn-MOF, which serves as a pivotal parameter for assessing the efficacy of these MOFs as antibiofilm agents. The IC_50_ of Zn-MOF is 0.01337 ± 0.007 mg mL^−1^, while the IC_50_ of Co-MOF is 0.01269 ± 0.007 mg mL^−1^. These results indicate that the high electrostatic interaction in Co-MOF could damage the bacterial community faster than in Zn-MOF, which possesses a small particle size with a high surface-to-volume ratio. Zn-MOF can penetrate the bacterial cell wall more readily due to its smaller size, while the higher oxidation potential of Co-MOF generates more electrostatic interactions with ROS that damage bacterial DNA and proteins.

This distinctive property renders Co-MOF a promising candidate for antibiofilm activity, promoting substantial electrostatic interactions with EPS that hinder biofilm formation. These results indicate that both Zn-MOF and Co-MOF exhibit comparable effectiveness against *S. aureus* ATCC 6538 in terms of biofilm inhibition. Furthermore, the exceptional redox stability exhibited by the Co-MOF makes it well suited for applications involving oxidation–reduction reactions. After assessing the antibiofilm activity, it was observed that the efficacy of the MOFs against biofilm was nearly the same, and transparent microwells were evident as the MOF concentration gradually increased to 0.160, 0.32, and 0.64 mg mL^−1^. The complete disappearance of the biofilm against *S. aureus* ATCC 6538 was observed at a concentration of 0.64 mg mL^−1^, which was equal to the MIC value against the *S. aureus* strains.

### 3.4 Antioxidant activities of the MOFs

One of the crucial considerations influencing the suitability of synthesized MOFs for biomedical applications under chronic inflammatory conditions is their biocompatibility in countering oxidative stress caused by FRs. These FRs can trigger inflammation, chronic diseases, and other harmful effects on living organisms. The DPPH assay was used to assess the antioxidant efficacy of MOFs, a commonly used technique for evaluating the antioxidant capacity of synthesised compounds, medicines, extracts, or biological sources. The antioxidant activity of the MOFs was shown by an adverse relationship between the violet color intensity of DPPH and the activity. The MOFs were assessed because of their ability to inhibit DPPH, and the % inhibition was calculated using eqn (2), as illustrated in Fig. 6S and Table 8S (ESI†). The findings shown in Fig. 9B clearly demonstrate the robust antioxidant effects of both Zn-MOF and Co-MOF at various concentrations. However, there exists a dissimilarity between the antioxidant properties of MOFs in terms of their IC_50_ values, which were calculated using a nonlinear regression fitting curve *via* GraphPad Prism 8 software. Specifically, the IC_50_ value for Co-MOF was determined to be 219.7 ± 7 μg mL^−1^, while Zn-MOF exhibited a higher IC_50_ value of 267.6 ± 7 μg mL^−1^, indicating that Co-MOF has a comparatively stronger antioxidant capacity. It is noteworthy that the MOFs exhibited lower free radical scavenging abilities when compared to the standard ascorbic acid (AA), which possessed an IC_50_ value of 6.464 ± 0.5 μg mL^−1^, as depicted in Fig. 9. The most notable scavenging activity was observed in AA, reaching 90.89% at a concentration of 16 μg mL^−1^.

Co-MOF demonstrated a scavenging activity of 52.17%, while Zn-MOF exhibited 40.83% at a concentration of 160 μg mL^−1^. Furthermore, an increase in MOF doses resulted in a proportional increase in antioxidant activity. The antioxidant activity of the MOFs appears to stem from two proposed mechanisms.^71^ First, the transfer of free electrons from various oxidation states of Co-MOF, containing Co^3+^ and Co^2+^ ions, facilitates the neutralization of free radicals, reducing their reactivity and harmful effects.^72^ Second, there is the transfer of free electrons from the oxygen atom of the MOFs to the free radicals on the nitrogen atoms in the DPPH. Notably, the observed high antioxidant capacity is particularly evident in materials with a high surface-to-volume ratio, as exemplified by Zn-MOF.^73^ There is a lack of comprehensive research on the antioxidant characteristics of MOFs. The findings of our study, however, show great promise and offer a valuable starting point for further investigation into the potential of Zn-MOF and Co-MOF as multifunctional materials. Therefore, it is essential to design and develop MOFs with inherent antibacterial, antibiofilm, and antioxidant properties to enhance their biocompatibility by optimizing their structures and exploring their potential as innovative solutions for combating oxidative stress-related issues. By enhancing biocompatibility and addressing oxidative stress concerns, MOFs hold promise for advancing various fields and improving human health and well-being. Both MOFs exhibited oxidant characteristics by providing oxidative stress to bacterial species and antioxidant behavior against free radical species. The dual properties of MOFs, acting as both oxidants and antioxidants, depend on factors such as the type of metal ions, organic ligands, and types of cells or molecules that contribute to the multifaceted behavior of the MOFs.^74^ After extensive reviews, some studies have shown that different kinds of MOFs can display antibacterial, antibiofilm, and antioxidant activities, as demonstrated in Table 4. These studies have illustrated that each MOF is suitable for one of the specified applications. More studies should be conducted to investigate the potential of MOFs for multifunctional applications in biomedical fields. The leakage of metals from the MOF frames was characterized using ICP analysis. Both Zn-MOF and Co-MOF were found to release metal ions at concentrations below the detection limit at the working pH (pH = 7.2). The leaching of zinc and cobalt ions was minimal, making it negligible for applications involving antibacterial, antioxidant, and antibiofilm activities. However, variations in pH can lead to the decomposition of the MOF frame, particularly in acidic or basic conditions. Metal ions can leak out of the MOF structure up to parts per billion (ppb) when the pH is basic, as shown in Table 9S (ESI†). Leaching tests were performed to determine how much Zn and Co ions were being released and to observe whether the MOFs could be used again, especially for their antioxidant properties in future uses. MOFs are susceptible to degradation under various environmental conditions, including changes in pH and heat exposure. Stability depends on metal ions, organic linkers, coordination bonds, and the pore environment.^75^ Enhancement strategies include using robust metal ions, rigid and symmetrical organic linkers; incorporating functional groups; and using solvothermal or hydrothermal synthesis methods, and post-synthetic treatments.^75^ These methods help minimize structural flexibility, enhance coordination bonds, and remove residual solvent molecules, thereby enhancing MOF stability.

**Table 4:** Antibacterial, antibiofilm, and antioxidant activity of various MOFs in the literature

MOFs	Organic linkers	Antioxidant activity (IC_50_)	Antibiofilm activity (IC_50_)	Antibacterial activity against	Ref.
NH_2_-MIL-125	2-Aminoterephthalic acid	—	*E. coli*	*E. coli*	33
			*S. aureus*	*S. aureus*	
			—		
Zr-MOF and Ti-MOF	2-Aminoterephthalic acid and silane surface modification	Animal study and plasma detection *via* DPPH˙	—	—	38
Zn-PDA	2,6-Pyridine dicarboxylic acid	—	—	*E. coli*	51
ZnO@ZIF-8	2-Methylimidazole	—	—	*E. coli* and *S. aureus*	66
Zn-MOF	4,4′-Bipyridine	—	—	*S. epidermidis*, *E. coli*	67
Calcium gallate MOFs (MIL-155 and MIL-156)	Gallic acid	Dichlorofluorescein diacetate (125 and 250 mg mL^−1^)	—	—	68
AgTAZ, ZIF-67, and Co-SIM-1	1,2,4-Triazole and 2-methylimidazole	—	—	*E. coli* CECT-4102 *Pseudomonas putida* CECT-4584, and *Saccharomyces cerevisiae* CECT-1170	69
Co-MOF@graphene oxide	1,4-Benzenedicarboxylic acid	*Via* DPPH˙		*E. coli* ATCC-25922 *S. aureus* ATCC-33591	70
Zn-MOF and Co-MOF	4,6-Diamino-2-pyrimidinethiol	DPPH˙ (0.2676 and 0.2197 mg mL^−1^)	Against *S. aureus* ATCC 6538 (0.01337 and 0.01269 mg mL^−1^)	*S. aureus* (clinic) *S. aureus* ATCC 6538 *P. aeruginosa* (clinic) *P. aeruginosa* ATCC 9027	This work

## 4. Conclusions

Multifunctioning MOFs designated as Zn-MOF and Co-MOF were synthesized using a cost-effective hydrothermal method. They underwent various thermal, electronic, and spectroscopic techniques. The MOFs revealed high efficacy in terms of their antibacterial, antioxidant, and antibiofilm activities. A comprehensive comparison of these MOFs highlighted their distinctive properties, emphasizing their significant antioxidant, antibacterial, and antibiofilm efficacies. The antibacterial assessments included MIC, MBC, and WDA against clinical isolates of *S. aureus*, and *P. aeruginosa*, as well as their standard strains. Zn-MOF showed better antibacterial activity than Co-MOF by applying the WDA method and IZD values. The MOFs demonstrated bactericidal activity against *S. aureus* strains, while they displayed bacteriostatic activity against *P. aeruginosa* strains based on MBC/MIC ratios. MOFs induce bacterial cell death through various detrimental mechanisms, such as ROS generation, electrostatic interaction, and lipid peroxidation. In addition, antibiofilm inhibition tests were performed on *S. aureus* ATCC 6538. The IC_50_ values for Zn-MOF and Co-MOF were found to be 0.01337 ± 0.007 mg mL^−1^ and 0.01269 ± 0.007 mg mL^−1^, respectively. This suggests that the strong electrostatic interaction in Co-MOF may lead to quicker damage to the bacterial community compared to Zn-MOF. The antioxidant abilities of Zn-MOF and Co-MOF were tested using the DPPH free radical scavenging protocol. Both MOFs showed robust antioxidant effects at various concentrations, but there was a difference in their antioxidant properties. Co-MOF demonstrated a stronger antioxidant capacity with an IC_50_ value of 219.7 ± 7 μg mL^−1^, while Zn-MOF had a higher IC_50_ value of 267.6 ± 7 μg mL^−1^, indicating the superior antioxidant performance of Co-MOF.

## Conflicts of interest

The author affirms that they have no conflicts to declare.

## Supplementary Material

RA-014-D4RA00545G-s001
