# Supplementary material for: Antibacterial, antibiofilm, and antioxidant activities of two novel metal–organic frameworks (MOFs) based on 4,6-diamino-2-pyrimidinethiol with Zn and Co metal ions as coordination polymers
Source: RSC Adv. 2024 Mar 18;14(13):9080–98. doi: 10.1039/d4ra00545g (PMC10945374; doi:10.1039/d4ra00545g)
Supplement: RA-014-D4RA00545G-s001 [file RA-014-D4RA00545G-s001.pdf]

Electronic Supplementary Material (ESI) for RSC Advances.
This journal is © The Royal Society of Chemistry 2024

Electronic Supplementary Materials

Antibacterial, antibiofilm, and antioxidant activities of two novel metal-organic frameworks (MOFs) based on 4,6-diamino-2-pyrimidinethiol with Zn and Co metal ions as coordination polymers.

Rebaz F. Hamarawf [a,b,*]

[a] Department of Chemistry, College of Science, University of Sulaimani, Sulaymaniyah city-Kirkuk Road, 46001, Kurdistan Region, Iraq

[b] Department of Medical Laboratory Science, Komar University of Science and Technology (KUST), Sulaymaniyah city-Qliasan St, 46002, Kurdistan Region, Iraq

Corresponding addresses: **rebaz.hamarawf@univsul.edu.iq**

Table 1S. XRD data were used to calculate microstrain and crystallite size based on FWHM using the Scherrer equation for Zn-MOF.

**Parameters**		**Peak position**	**FWHM**	**Crystallite Size (nm)**	**Microstrain ($\varepsilon x10^{-3}$)**
**K**	**λ (Å)**	**2θ (°)**	**β (°)**	$D = \frac{K\lambda}{\beta\cos\theta}$	$\varepsilon = \frac{\beta}{4\tan\theta}$
**0.94**	**1.5406**	**28.595**	**0.1968**	**43.51**	**3.3694**
		**31.6015**	**0.2952**	**29.21**	**4.5517**
		**34.3561**	**0.1968**	**44.13**	**2.7778**
		**36.1735**	**0.3936**	**22.18**	**5.2585**
		**47.6945**	**0.3444**	**26.34**	**3.3995**
		**56.6322**	**0.1968**	**47.89**	**1.5937**
		**62.7646**	**0.492**	**19.75**	**3.5194**
		**67.8718**	**0.246**	**40.65**	**1.5952**
		**77.159**	**0.984**	**10.79**	**5.3823**
				**Average**	**Average**
				**31.61**	**3.49**

Table 2S. XRD data were used to calculate microstrain and crystallite size based on FWHM using the Scherrer equation for Co-MOF.

**Parameters**		**Peak position**	**FWHM**	**Crystallite Size (nm)**	**Microstrain ($\varepsilon x10^{-3}$)**
**K**	**λ (Å)**	**2θ (°)**	**β (°)**	$D = \frac{K\lambda}{\beta\cos\theta}$	$\varepsilon = \frac{\beta}{4\tan\theta}$
**0.94**	**1.5406**	10.6892	0.492	16.94	22.9471
		15.274	0.984	8.5	34.0248
		17.3764	0.984	8.53	28.0969
		23.9168	0.8856	9.58	18.2446
		29.7828	0.984	8.73	16.1459
		32.9431	0.7872	10.99	11.6169
		34.7355	0.7872	11.04	10.9821
		36.6024	0.7872	11.1	10.3852
		38.381	0.8856	9.92	11.1023
		41.6333	0.7872	11.28	9.0343
		44.7018	0.09	99.68	0.9551
		47.7623	0.09	100.82	0.887
		52.4363	0.9348	9.89	8.2827

		59.424	0.09	106.15	0.6881
		61.7985	0.09	107.44	0.6562
		67.6948	0.7872	12.69	5.1217
		73.4602	0.09	115.03	0.5263
		76.8901	0.09	117.71	0.4947
		**Average**	**Average**	**43.11**	**10.56**

Table 3S. Antibiotics susceptibility test based on Kirby-Bauer test method used for all the bacteria.

**Antibiotics**	**IZD of antibiotics against the following antibiotics ±2 mm**			
	S. aureus (Clinic)	S. aureus (ATCC 6538)	*P. aeruginosa* (Clinic)	*P. aeruginosa* (ATCC 9027)
Vancomycin (VA)	16 mm	16 mm	0	0
Doxycycline (Do)	0	23 mm	0	0
Metronidazole (MET)	0	0	8 mm	0
Amikacin (AK)	0	15 mm	0	13 mm
Clarithromycin (CLR)	12 mm	28 mm	9 mm	9 mm
Meropenem (MRP)	0	21 mm	0	16 mm
Erythromycin (E)	8 mm	24 mm	7 mm	7 mm
Ceftriaxone (CTR)	0	20 mm	0	16 mm
Aztreonam (AT)	0	7 mm	0	21 mm
Gentamicin (CN)	0	16 mm	0	15 mm
Nalidixic acid (ND)	8 mm	12	0	0
Nitrofurantoin (NIT or F)	14 mm	15 mm	0	0
Amoxicillin/clavulanic acid (AMC)	0	25 mm	0	0
Oxacillin (OX)	0	21 mm	0	0
Ampicillin (AMP)	7 mm	33 mm	0	0
Rifampicin (RA)	10 mm	21 mm	0	0
Azithromycin (AZM)	9 mm	18 mm	21 mm	9 mm
Levofloxacin (LE)	12 mm	22 mm	0	21 mm
Cefotaxime/Clavulanic acid (CEC)	7 mm	28 mm	0	29 mm

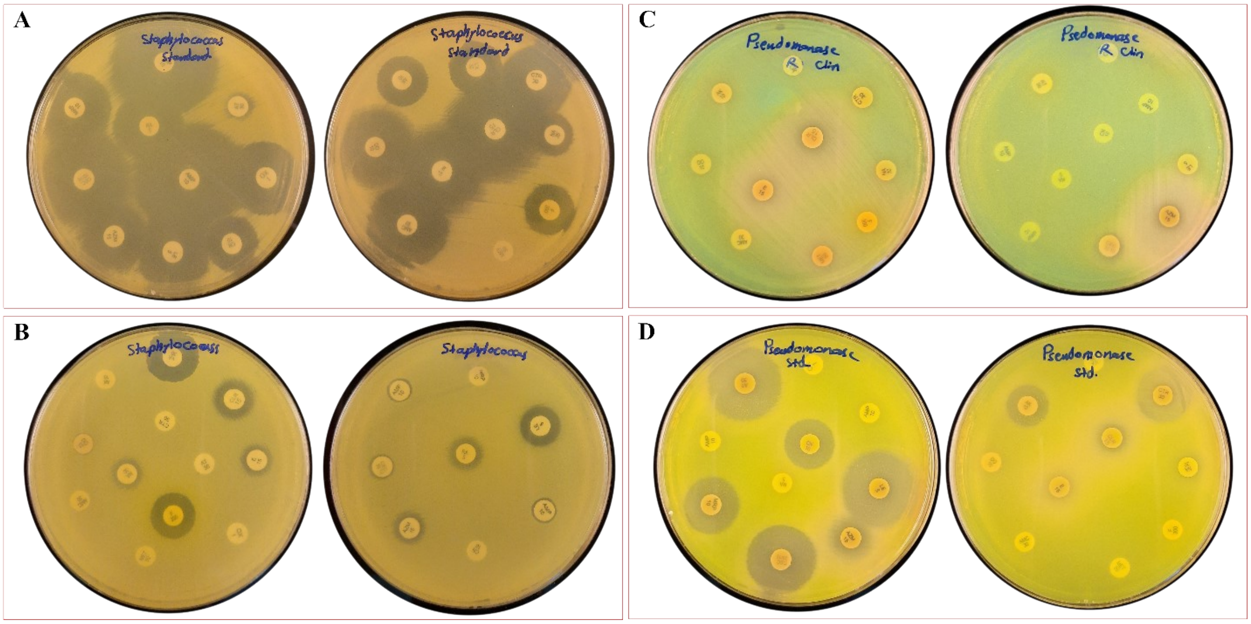

Fig. 1S Disc diffusion assay for antibiotic susceptibility testing using the Kirby-Bauer method against the following bacterial strains: (A) *Staphylococcus aureus* ATCC 6538, (B) clinical isolates of *Staphylococcus aureus*, (C) Clinical isolate of *Pseudomonas aeruginosa*, and (D) *Pseudomonas aeruginosa* ATCC 9027.

Table 4S. OD of the Zn-MOF against clinical isolates of *Staphylococcus aureus* (A & B) and *Pseudomonas aeruginosa* (E & F), as well as their standard strains (*Staphylococcus aureus* ATCC 6538 (C & D) and *Pseudomonas aeruginosa* ATCC 9027 (G & H)).

ppm:	10	20	40	80	160	320	640	1260	2560	+ Ve	- Ve	- Ve	
	1	2	3	4	5	6	7	8	9	10	11	12	
A	0.073	0.062	0.071	0.066	0.059	0.061	0.049	0.049	0.049	0.081	0.059	0.038	600
	0.073	0.062	0.071	0.066	0.059	0.061	0.049	0.049	0.049	0.081	0.059	0.038	600[2]
B	0.071	0.061	0.068	0.067	0.059	0.059	0.043	0.05	0.05	0.081	0.058	0.038	600
	0.071	0.061	0.068	0.067	0.059	0.059	0.043	0.05	0.05	0.081	0.058	0.038	600[2]
C	0.065	0.066	0.068	0.065	0.049	0.049	0.037	0.043	0.043	0.125	0.062	0.035	600
	0.065	0.066	0.068	0.065	0.049	0.049	0.037	0.043	0.043	0.125	0.062	0.035	600[2]
D	0.059	0.071	0.063	0.058	0.05	0.047	0.053	0.045	0.045	0.129	0.061	0.038	600
	0.059	0.071	0.063	0.058	0.05	0.047	0.053	0.045	0.045	0.129	0.061	0.038	600[2]
E	0.046	0.041	0.05	0.04	0.043	0.042	0.04	0.049	0.041	0.091	0.06	0.036	600
	0.046	0.041	0.05	0.04	0.044	0.042	0.04	0.049	0.04	0.093	0.06	0.036	600[2]
F	0.072	0.043	0.042	0.04	0.044	0.046	0.049	0.042	0.049	0.092	0.062	0.037	600
	0.072	0.043	0.042	0.04	0.044	0.046	0.049	0.042	0.049	0.093	0.062	0.037	600[2]
G	0.093	0.081	0.039	0.037	0.05	0.043	0.04	0.043	0.04	0.093	0.065	0.044	600
	0.093	0.081	0.039	0.037	0.05	0.043	0.04	0.043	0.041	0.092	0.065	0.044	600[2]
H	0.046	0.042	0.047	0.043	0.046	0.042	0.043	0.045	0.043	0.093	0.08	0.048	600
	0.045	0.042	0.047	0.043	0.046	0.042	0.043	0.045	0.043	0.093	0.08	0.048	600[2]

Table 5S. OD of the Co-MOF against clinical isolates of *Staphylococcus aureus* (A & B) and *Pseudomonas aeruginosa* (E & F), as well as their standard strains (*Staphylococcus aureus* ATCC 6538 (C & D) and *Pseudomonas aeruginosa* ATCC 9027 (G & H)).

ppm:	10	20	40	80	160	320	640	1260	2560	+ Ve	- Ve	- Ve	
	1	2	3	4	5	6	7	8	9	10	11	12	
A	0.091	0.093	0.096	0.099	0.091	0.075	0.042	0.05	0.055	0.087	0.047	0.037	1:600
	0.091	0.092	0.096	0.099	0.091	0.075	0.042	0.051	0.055	0.086	0.047	0.037	2:600
B	0.087	0.088	0.105	0.089	0.089	0.075	0.046	0.047	0.057	0.094	0.044	0.037	1:600
	0.087	0.088	0.105	0.089	0.089	0.075	0.046	0.047	0.057	0.095	0.044	0.037	2:600
C	0.14	0.152	0.087	0.131	0.077	0.064	0.048	0.074	0.047	0.133	0.044	0.036	1:600
	0.14	0.152	0.087	0.131	0.077	0.065	0.048	0.073	0.047	0.132	0.044	0.036	2:600
D	0.141	0.134	0.077	0.13	0.073	0.067	0.046	0.072	0.046	0.129	0.051	0.037	1:600
	0.142	0.135	0.077	0.13	0.073	0.067	0.046	0.073	0.046	0.129	0.051	0.038	2:600
E	0.091	0.053	0.052	0.04	0.072	0.042	0.045	0.047	0.052	0.092	0.046	0.036	1:600
	0.091	0.054	0.052	0.04	0.072	0.042	0.046	0.047	0.052	0.092	0.046	0.036	2:600
F	0.097	0.065	0.055	0.042	0.068	0.042	0.044	0.046	0.052	0.093	0.047	0.037	1:600
	0.097	0.064	0.055	0.043	0.068	0.042	0.044	0.047	0.053	0.093	0.047	0.037	2:600
G	0.091	0.048	0.049	0.041	0.042	0.043	0.041	0.041	0.043	0.094	0.046	0.039	1:600
	0.091	0.048	0.049	0.041	0.041	0.044	0.042	0.042	0.043	0.094	0.046	0.039	2:600
H	0.096	0.048	0.048	0.042	0.041	0.04	0.049	0.043	0.045	0.093	0.048	0.037	1:600
	0.096	0.049	0.048	0.041	0.042	0.04	0.049	0.044	0.045	0.093	0.048	0.037	2:600

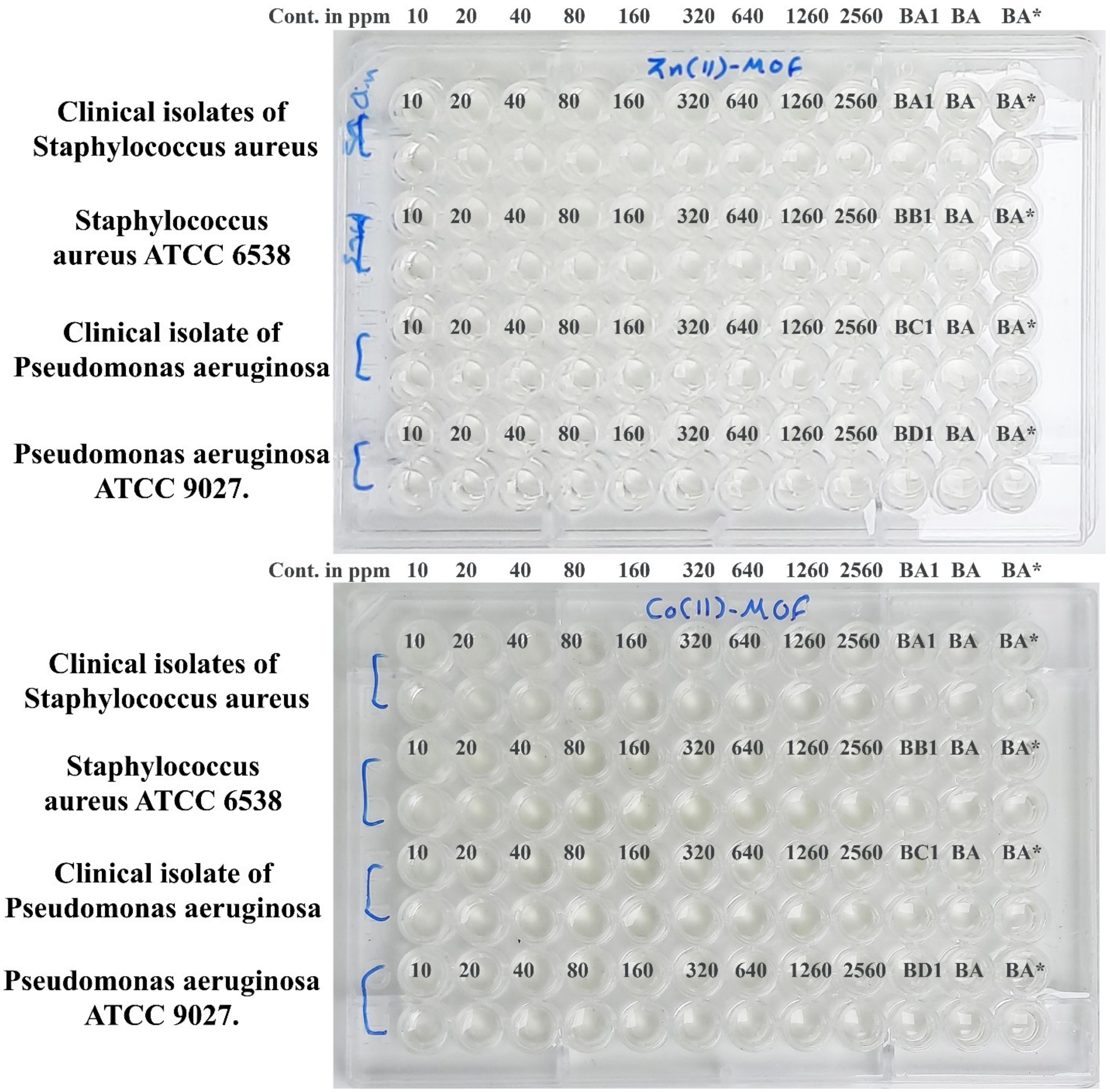

Fig. 2S The 96-well plate contained a series of concentrations of MOFs, which were used against four different bacterial strains. The plate was read at 600 nm.

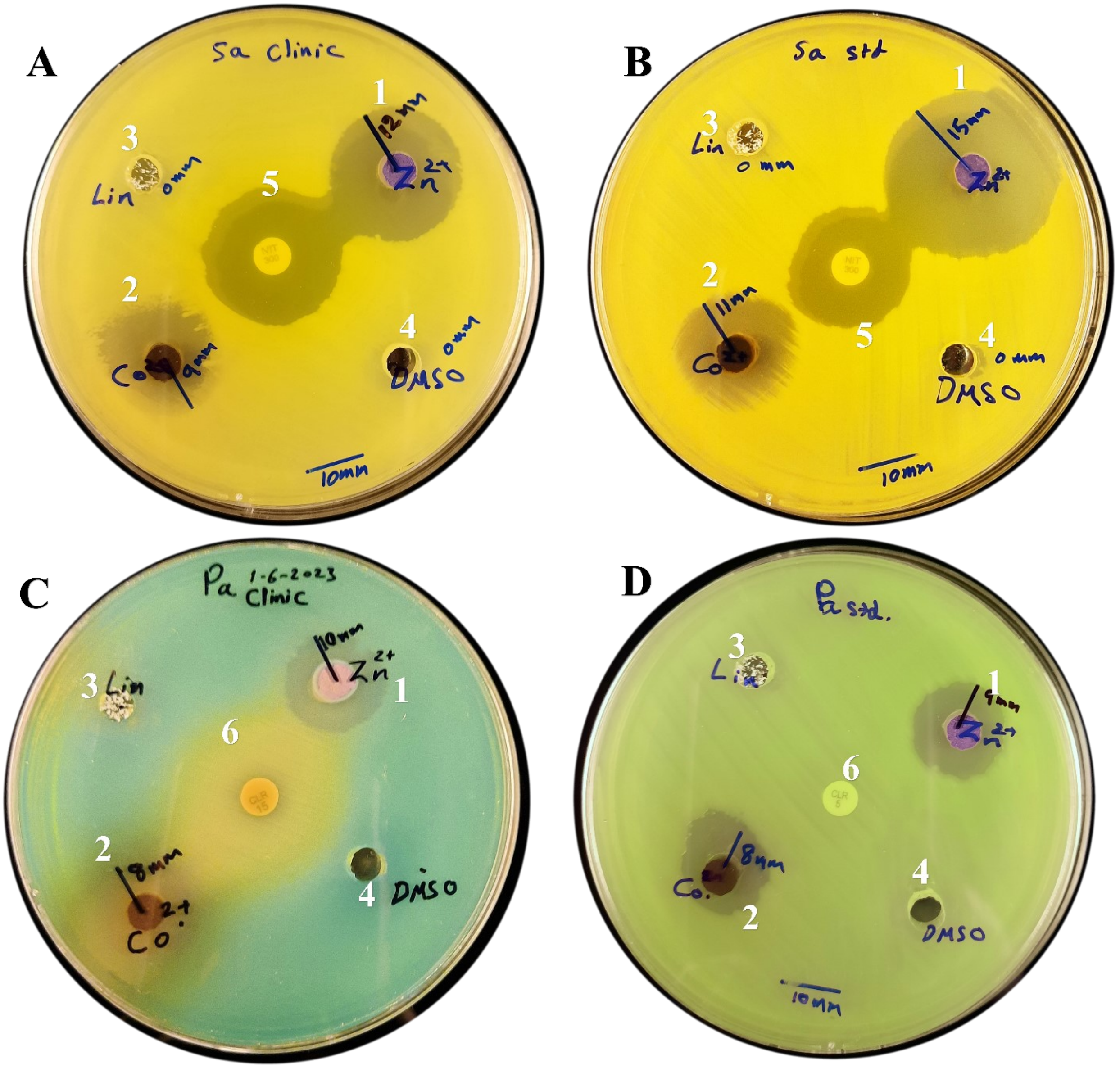

Fig. 3S demonstrates the second set of the antimicrobial activity against clinical isolates of (A) *Staphylococcus aureus* and (C) *Pseudomonas aeruginosa*, as well as their standard strains (B) *Staphylococcus aureus* ATCC 6538 and (D) *Pseudomonas aeruginosa* ATCC 9027. The different samples tested were: 1) Zn-MOF, 2) Co-MOF, 3) Organic linker, 4) DMSO, 5) Ceftriaxone (CTR), and 6) Clarithromycin (CLR).

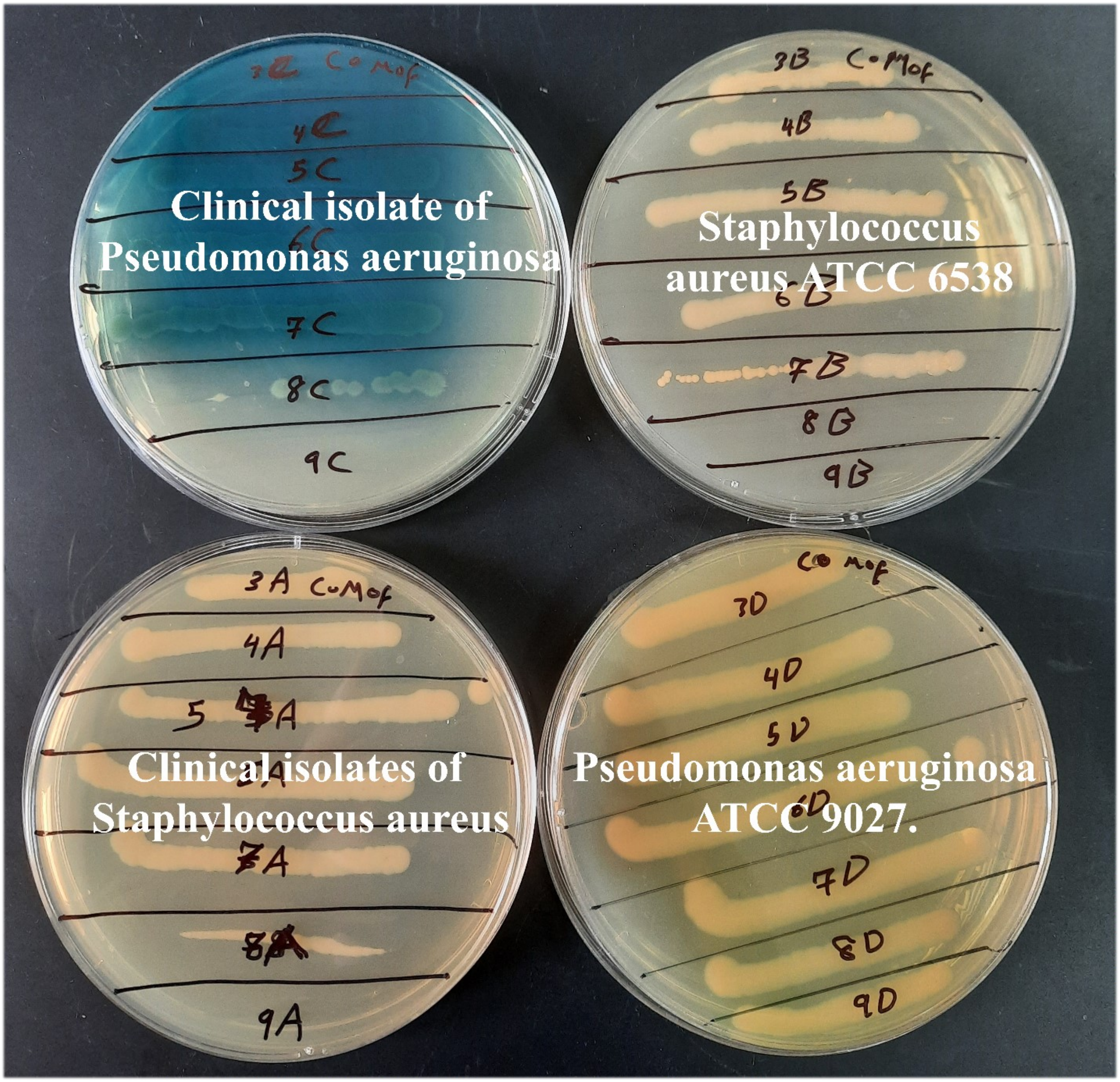

Fig. 4S MBC assay of the Co-MOF against four bacterial species.

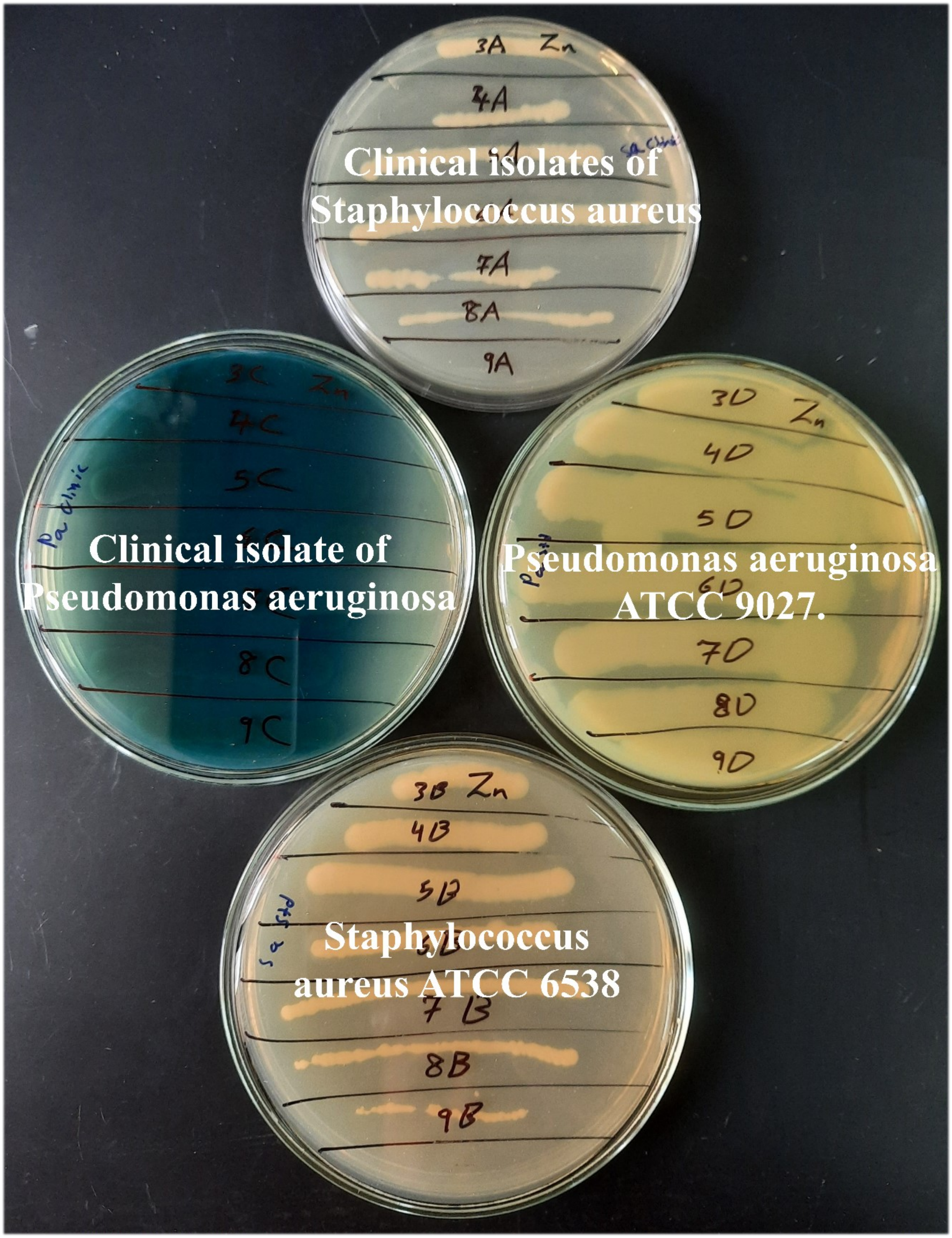

Fig. 5S MBC assay of the Zn-MOF against four bacterial species.

Table 6S. OD and percentage inhibition of the biofilm provided by the Zn-MOF.

**Concentration of control (DPPH)**	**Concentration of Zn(II) MOF**	**OD reading 1 Zn(II)**	**OD reading 2**	**OD of Zn(II)MOF2**	**% of inhibition of Zn-MOF= ((ODuntreated -ODtreated) /ODuntreated) x 100**
0.1% Crystal violet	10 ppm	0.099	0.1	0.0595	27.43902439
Wavelength 595 nm	20 ppm	0.066	0.067	0.0265	67.68292683
$OD_{Untreated}$	40 ppm	0.054	0.054	0.014	82.92682927
0.122	80 ppm	0.046	0.045	0.0055	93.29268293
OD_{Blank}	160 ppm	0.043	0.044	0.0035	95.73170732
0.04	320 ppm	0.043	0.044	0.0035	95.73170732
$OD_{Untreated}$ - OD_{Blank}	640 ppm	0.04	0.041	0.0005	99.3902439
0.082	1280 ppm	0.041	0.041	0.001	98.7804878
**Zn-MOF**	2560 ppm	0.04	0.04	0	100

Table 7S. OD and percentage inhibition of the biofilm provided by the Co-MOF.

**Concentration of control (DPPH)**	**OD of Co(II)MOF**	**OD reading 1 Co(II)**	**OD reading 2 Co(II)**	**OD of Co(II)**	**% of inhibition of Co-MOF= ((ODuntreated -ODtreated) /ODuntreated) x 100**
0.1% Crystal violet	10 ppm	0.097	0.099	0.058	29.26829268
Wavelength 595 nm	20 ppm	0.067	0.065	0.026	68.29268293
$OD_{Untreated}$	40 ppm	0.056	0.053	0.0145	82.31707317
0.122	80 ppm	0.048	0.049	0.0085	89.63414634
OD_{Blank}	160 ppm	0.047	0.047	0.007	91.46341463
0.04	320 ppm	0.045	0.046	0.0055	93.29268293
$OD_{Untreated}$ - OD_{Blank}	640 ppm	0.043	0.044	0.0035	95.73170732
0.082	1280 ppm	0.041	0.041	0.001	98.7804878
**Co-MOF**	2.560 ppm	0.04	0.041	0.0005	99.3902439

Table 8S. Absorbance and percentage inhibition of DPPH provided by the MOFs.

**Concentration of control (DPPH)**	**Concentration of Co-MOF**	**Concentration of Zn-MOF**	**Absorbance of Zn-MOF**	**Absorbance of Co-MOF**	**% inhibition of Zn-MOF =**	**% inhibition of Co-MOF= ((A0**

					((A0 - A1) /A0) x 100	- A1) /A0) x 100
0.1 mM (1 mL DPPH + 1 mL (DMSO)	10 ppm	10 ppm	0.513	0.509	3.024574669	3.780718336
Wavelength: 517 nm	20 ppm	20 ppm	0.512	0.502	3.213610586	5.103969754
Ascorbic acid: 100 ppm	40 ppm	40 ppm	0.477	0.46	9.829867675	13.04347826
Abs std as blank 0.021	80 ppm	80 ppm	0.451	0.42	14.74480151	20.60491493
	160 ppm	160 ppm	0.313	0.253	40.83175803	52.17391304
	320 ppm	320 ppm	0.229	0.216	56.71077505	59.16824197
	640 ppm	640 ppm	0.105	0.145	80.15122873	72.58979206
	1280 ppm	1280 ppm	0.081	0.052	84.68809074	90.17013233
	2560 ppm	2560 ppm	0.029	0.022	94.51795841	95.84120983

Table 9S. Zinc and Cobalt Ions Leaching from MOFs under Various pH Conditions.

**0.15 g (15000 ppm) of the MOFs were dissolved in a 15 mL mixture of DMSO: DDW (1:1)**			
**MOFs**	**Zn^{2+} & Co^{2+} in ppb (pH = 7)**	**Zn^{2+} & Co^{2+} in ppb (pH = 3)**	**Zn^{2+} & Co^{2+} in ppb (pH = 11)**

Zn-MOF	BDL	45.5	134.6
Co-MOF	BDL	32.1	122.1
**BDL stands for Below Detection Limit**			

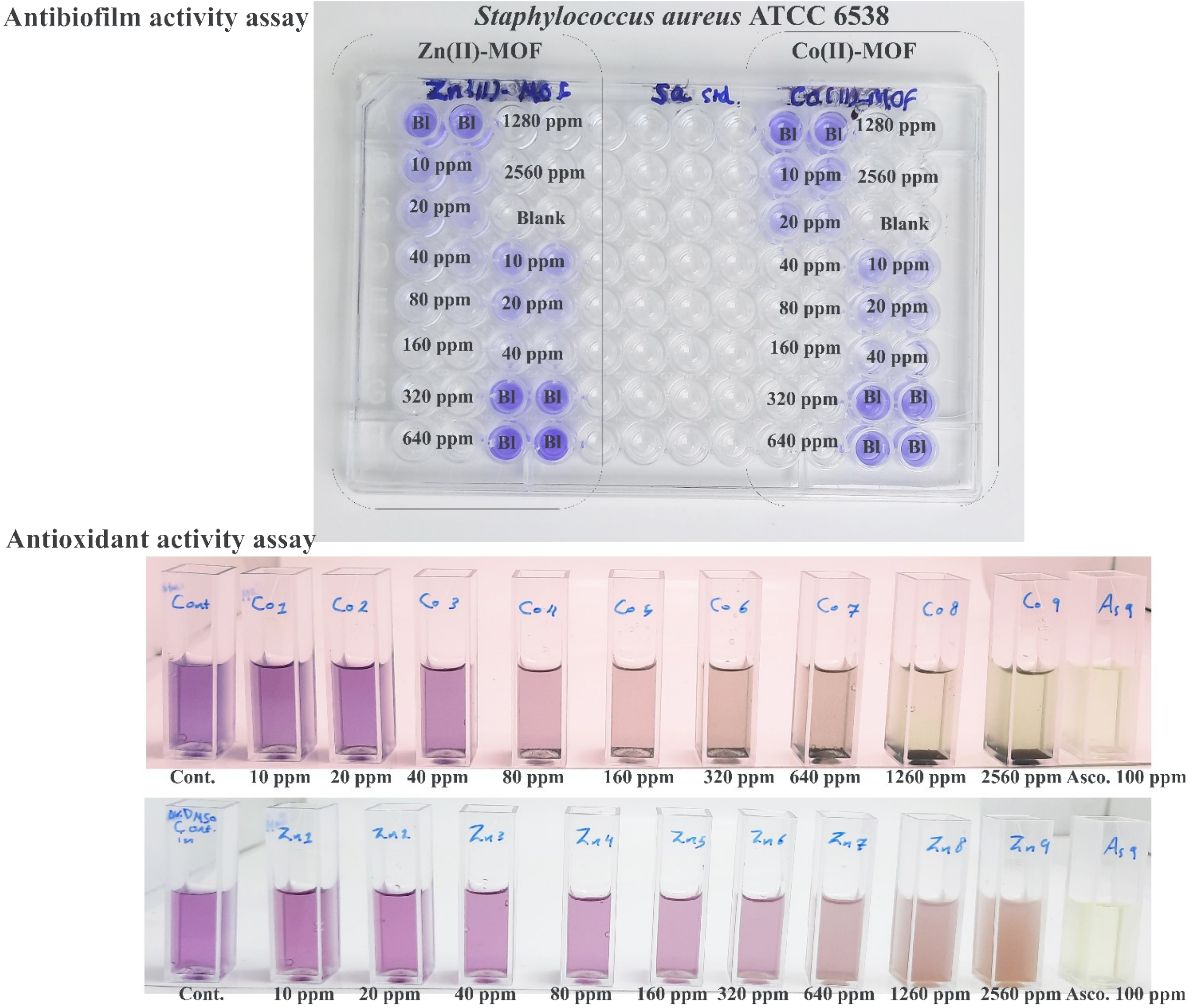

Fig. 6S Antibiofilm and an antioxidant assay of the MOFs.

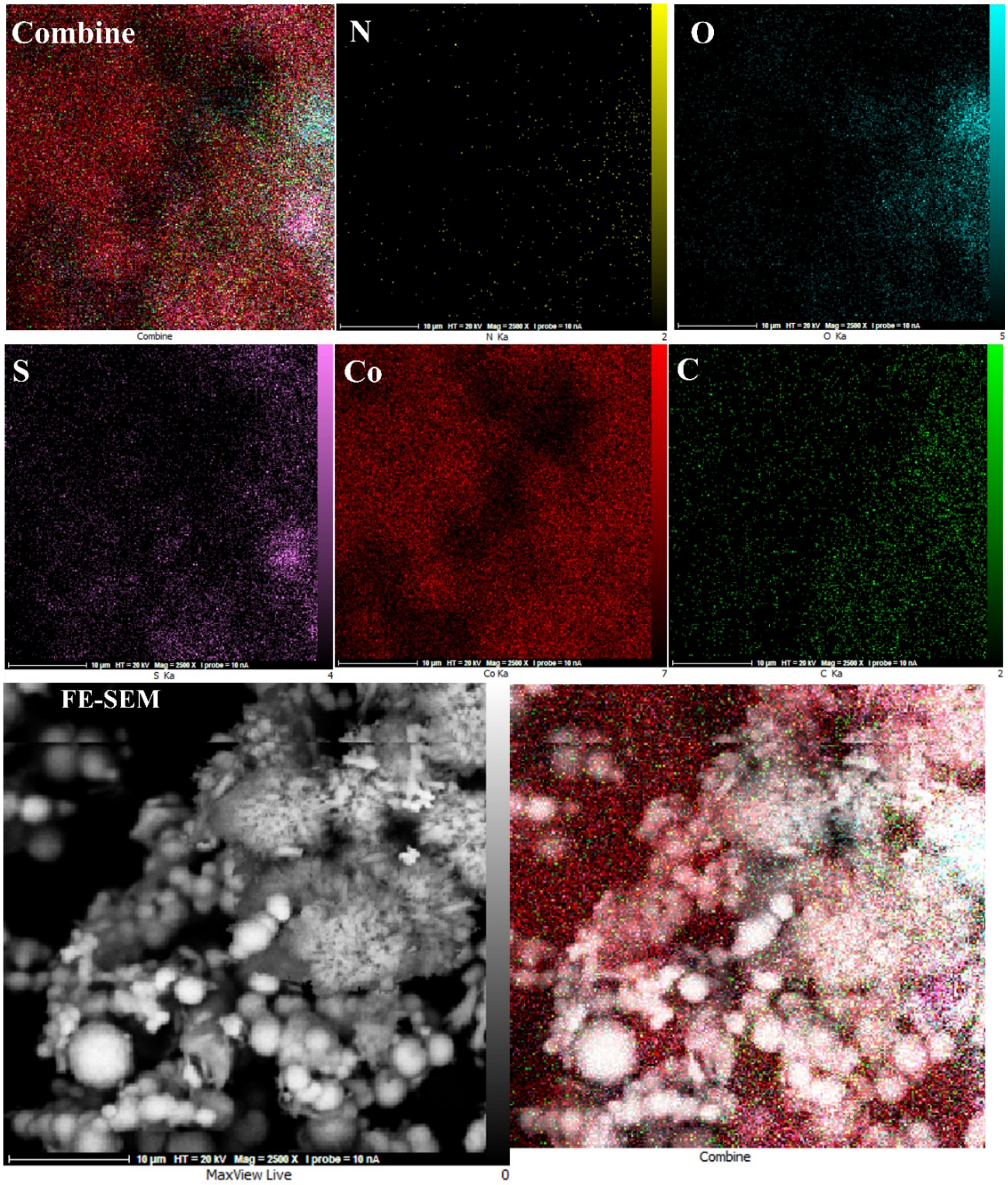

Fig. 7S EDX elemental surface mapping of synthesized Co-MOF.

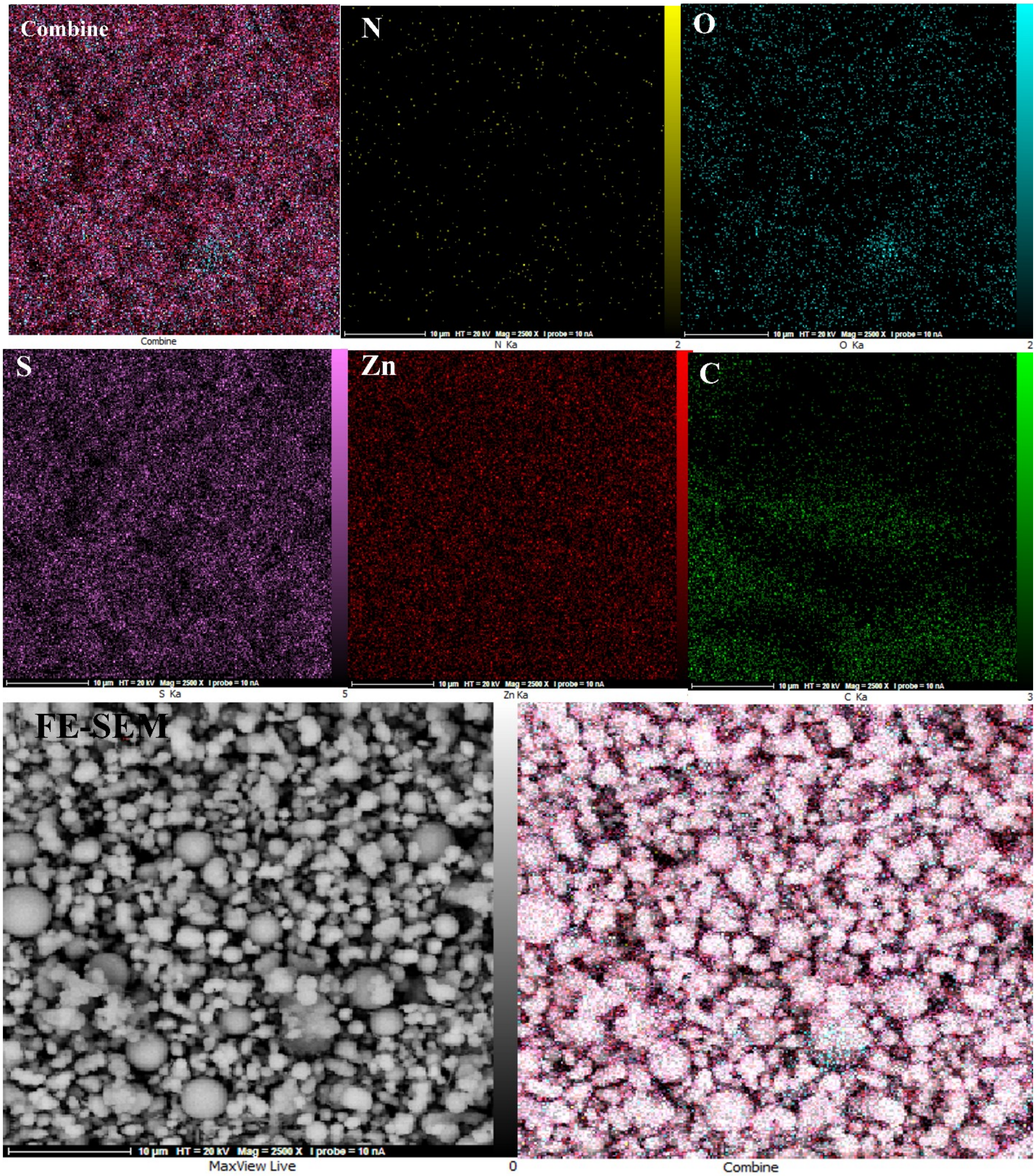

Fig. 8S EDX elemental surface mapping of synthesized Zn-MOF.
